# Screening and identification of angiogenesis-related genes as potential novel prognostic biomarkers of hepatocellular carcinoma through bioinformatics analysis

**DOI:** 10.18632/aging.203260

**Published:** 2021-07-12

**Authors:** Zili Zhen, Zhemin Shen, Yanmei Hu, Peilong Sun

**Affiliations:** 1 Center for Tumor Diagnosis and Therapy, Jinshan Hospital, Fudan University, Shanghai 201508, China; 2 Department of General Surgery, Jinshan Hospital, Fudan University, Shanghai 201508, China; 3 Department of Surgery, Shanghai Medical College, Fudan University, Shanghai 200032, China; 4 Department of Paediatrics, the Second Hospital of Jilin University, Changchun 130041, Jilin, China

**Keywords:** hepatocellular carcinoma, angiogenesis, gene signature, prognosis, bioinformatics analysis

## Abstract

Hepatocellular carcinoma (HCC) is a malignant tumor with high morbidity and mortality, which makes the prognostic prediction challenging. Angiogenesis appears to be of critical importance in the progression and metastasis of HCC. Some of the angiogenesis-related genes promote this process, while other anti-angiogenesis genes suppress tumor growth and metastasis. Therefore, the comprehensive prognostic value of multiple angiogenesis-related genes in HCC needs to be further clarified. In this study, the mRNA expression profile of HCC patients and the corresponding clinical data were acquired from multiple public databases. Univariate Cox regression analysis was utilized to screen out differentially expressed angiogenesis-related genes with prognostic value. A multigene signature was established with the least absolute shrinkage and selection operator Cox regression in the Cancer Genome Atlas cohort, and validated through an independent cohort. The results suggested that a total of 16 differentially expressed genes (DEGs) were associated with overall survival (OS) and a 7-gene signature was constructed. The risk score of each patient was calculated using this signature, the median value of which was used to divide these patients into a high-risk group and a low-risk group. Compared with the low-risk group, the patients in the high-risk group had a poor prognosis. The risk score was an independent predictor for OS through multivariate Cox regression analysis. Then, unsupervised learning was used to verify the validity of this 7-gene signature. A nomogram by further integrating clinical information and the prognostic signature was utilized to predict prognostic risk and individual OS. Functional enrichment analyses demonstrated that these DEGs were enriched in the pathways of cell proliferation and mitosis, and the immune cell infiltration was significantly different between the two risk groups. In summary, a novel angiogenesis-related genes signature could be used to predict the prognosis of HCC and for targeted therapy.

## INTRODUCTION

Primary liver cancer is a malignant tumor with the sixth morbidity rate and the fourth mortality rate in the world. The most common subtype is hepatocellular carcinoma (HCC), which accounts for 75%–85% of cases [1, 2]. The benefits of a vaccine against the hepatitis virus, and the improvement of diagnostic measures contribute to a reduction in the morbidity of HCC. Otherwise, the mortality rate is still high due to the limitations of surgical resection, orthotopic liver transplantation, or local percutaneous tumor ablation, especially for patients with advanced HCC or Child-Pugh class C cirrhosis [3]. The high heterogeneity of HCC and the complex impact of many factors in the advancing process make prognosis prediction challenging [4, 5]. To improve the prognosis of HCC patients, hence, identifying biomarkers for prognostic prediction and treatment of HCC is critical.

Angiogenesis occurs in some physiological processes involving tissue repair, proliferation, and remodeling, mainly including wound healing, embryonic development, and various pathophysiological processes, such as cancer, inflammation, and atherosclerosis [6–9]. Coordinated by angiopoietin and angiostatin produced by angiogenesis-related genes, angiogenesis mainly involves the following steps: production of angiogenic growth factors, degradation of the basement membrane, proliferation, migration, luminal formation, differentiation, and maturation characterized by endothelial cells, and regulation of vascular supporting cells [10].

As a solid tumor rich in blood vessels, HCC has obvious vascular proliferation and abnormal blood vessels, whose growth, invasion, and metastasis are partly caused by tumor angiogenesis. Of the numerous angiogenesis pathways, the VEGF/VEGFR receptor signaling pathway has been verified as the target of HCC precision medicine, which is targeted to inhibit angiogenesis and achieve the treatment for advanced HCC by sorafenib [11–13]. Apart from this, some of the other genes have also been proposed as biomarkers or modulators of angiogenesis. Certain genes, such as HIF1A, TMPRSS4, and SDF-1, regulate angiogenesis positively, which plays a vital part in promoting HCC progression and metastasis [14–16]. Undeniably speaking, some angiogenesis-related genes, including ANGPTL1 and EYA4, inhibit the angiogenesis and subsequent deterioration of HCC [17, 18]. Nonetheless, it remains unknown about the relation between the angiogenesis-related genes and the prognosis of HCC patients.

Different angiogenesis-related genes play a role in promoting or suppressing cancer respectively, with the results showing that a single gene, promoting or inhibiting angiogenesis, cannot be adequately predicted prognosis. Thus, a model integrating multiple angiogenesis-related genes may have a preliminary judgment on the prognosis of HCC patients to a certain extent. First off, we gained transcriptome data and clinical information in multiple datasets. After that, we screened out the angiogenesis-related differentially expressed genes (DEGs) with prognostic value in the Cancer Genome Atlas (TCGA) dataset through the univariate Cox regression analysis, thereby constructing a multigene prognostic signature, and verified it in the International Cancer Genome Consortium (ICGC) cohort. This model was significantly related to several clinical characteristics (grade, stage, T classification and tumor status) of HCC patients. A nomogram by further integrating clinical information and the prognostic gene signature was utilized to predict prognostic risk and individual survival rate. Finally, we explored the distinctions in the critical functions, signaling pathways and immune infiltration between high-risk and low-risk groups using Kyoto Encyclopedia of Genes and Genomes (KEGG), Gene Ontology (GO), and single sample Gene Set Enrichment Analysis (ssGSEA) to definite the underlying mechanisms. The immunophenoscore (IPS) gap between the two groups also indicated that the model had a deep relationship with immunotherapy.

## RESULTS

### Clinical characteristics of HCC patients

**Figure 1:**
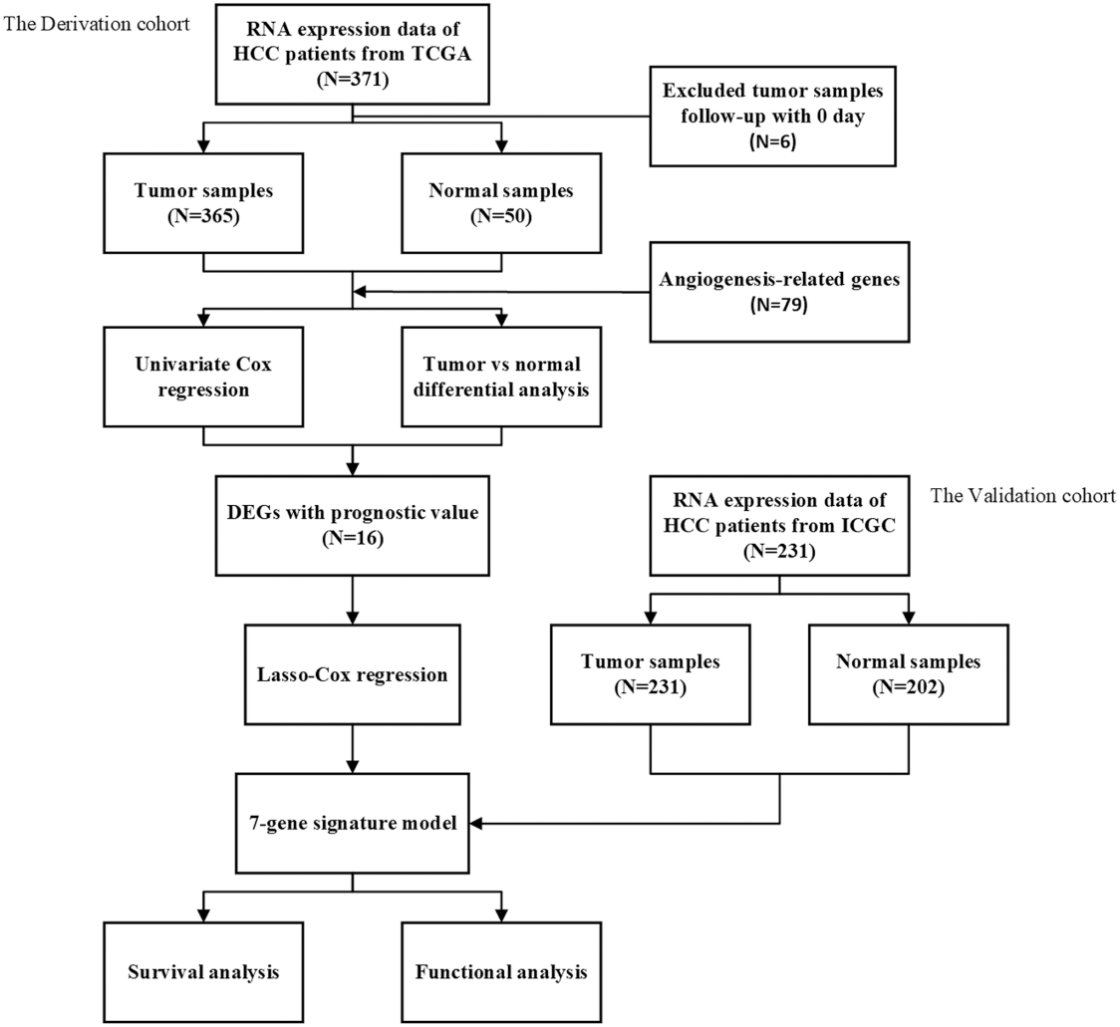
The flow chart of data collection and analyses.

Figure 1 is a flow chart of this research. After excluding patients with a follow-up time of 0 days or lack of clinical data, our study finally included 365 HCC patients from the TCGA dataset as a training set, and 231 HCC patients from the ICGC dataset as a validation set. The demographic and specific clinical features of the HCC samples in both cohorts were listed in Table 1.

**Table 1:** The demographic and clinical characteristics of HCC patients in this study.

	**TCGA cohort**	**ICGC cohort**
**No. of patients**	365	231
**Age (median, range)**	60 (16–90)	67 (31–89)
**Gender (%)**		
Female	119 (32.6%)	61 (26.4%)
Male	246 (67.4%)	170 (73.6%)
**Grade (%)**		
G1	55 (15.1%)	–
G2	175 (47.9%)	–
G3	118 (32.3%)	–
G4	12 (3.3%)	–
unknow	5 (1.4%)	–
**Stage (%)**		
Stage I	170 (46.6%)	36 (15.6%)
Stage II	84 (23.0%)	105 (45, 5%)
Stage III	83 (22.7%)	71 (30.7%)
Stage IV	4 (1.1%)	19 (8.2%)
unknow	24 (6.6)	0 (0.0%)
**Survival status**		
OS days (median, range)	812 (1–3675)	812 (10–2160)
Death (%)	130 (35.6%)	42 (18.2%)

### Identification of prognostic angiogenesis-related DEGs in the TCGA dataset

**Figure 2:**
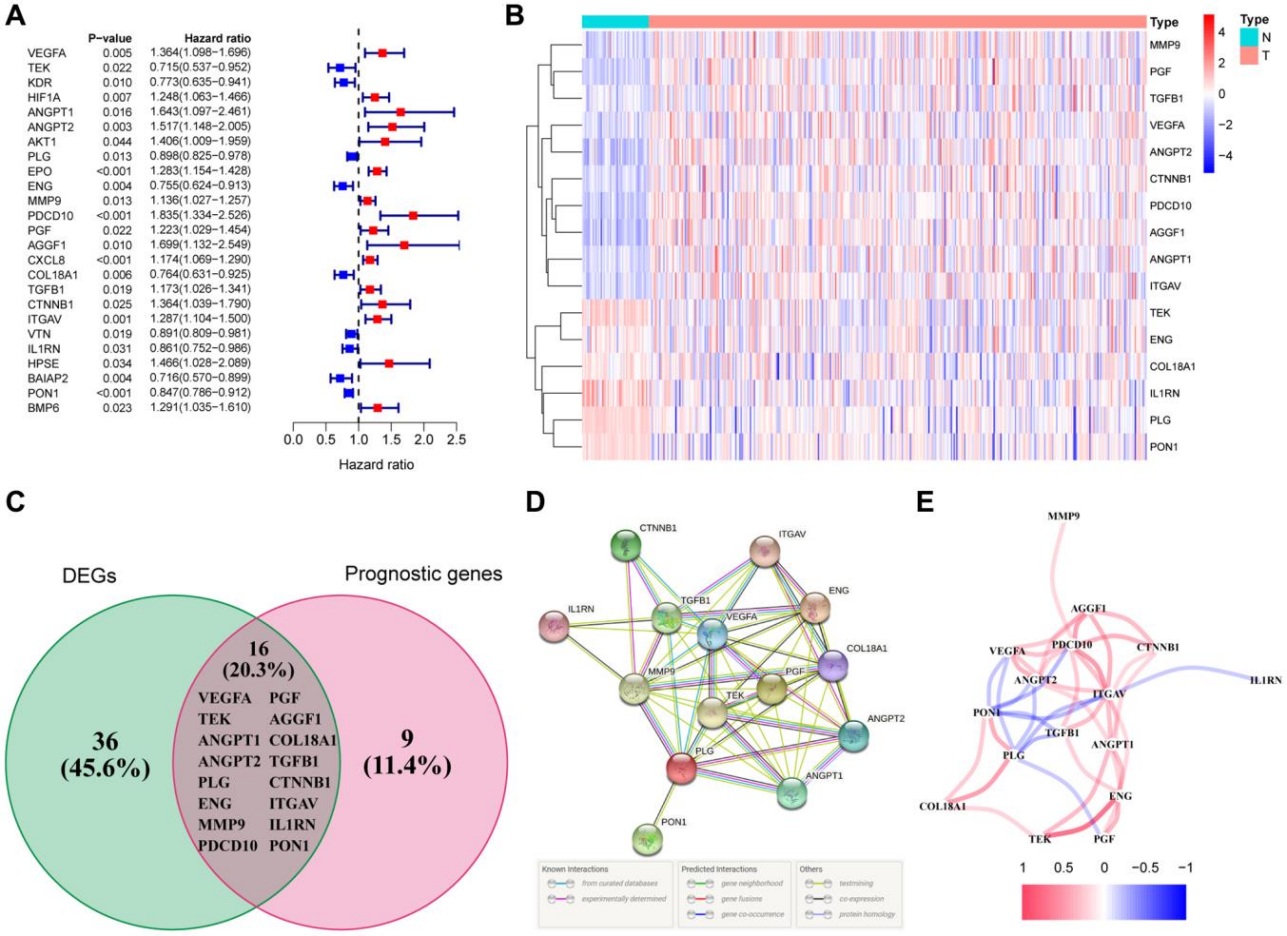
**Identification of the differentially expressed angiogenesis-related genes with prognostic value in the TCGA cohort.** (**A**) Forest plots showing the results of the univariate Cox regression analysis between gene expression and OS. (**B**) The heatmap showing the expression of 16 overlapping genes in tumor tissues. (**C**) The DEGs with prognostic value were obtained by the intersection of the two groups of genes in the venn diagram. (**D**) The PPI network downloaded from the STRING database indicated the interactions among the candidate genes. (**E**) The correlation network of the candidate genes.

We screened out 52 genes from 79 angiogenesis-related genes with significant differential expression between adjacent normal tissues and tumor tissues (Supplementary Table 1). With the help of univariate Cox regression analysis, 25 genes were closely related to the prognosis of HCC (Figure 2A). By taking the intersection of both sets of genes, it was clear that 16 DEGs were tightly bound to the overall survival (OS) of HCC patients (Figure 2B, 2C). MMP9, PGF, TGFB1, VEGFA, ANGPT2, CTNNB1, PDCD10, AGGF1, ANGPT1, ITGAV had a higher hazard ratio, and were highly expressed in HCC tissues, indicating that the expression of these genes could promote the secretion of angiogenic factors, and angiogenesis was also accelerated with abnormalities of tumor blood vessels, leading to rapid tumor development and poor prognosis. On the contrary, the hazard ratio of TEK, ENG, COL18A1, IL1RN, PLG, and PON1 was less than 1. Moreover, these 6 genes were highly expressed in normal tissues, and had the function of normalizing blood vessels. High expression of these genes is conducive to prognosis. VEGFA and TEK were clearly identified as the core genes according to the interaction network (Figure 2D). Meanwhile, the correlation diagram based on these angiogenesis-related genes was exhibited in Figure 2E.

### Establishment of a prognostic signature using the TCGA dataset

**Figure 3:**
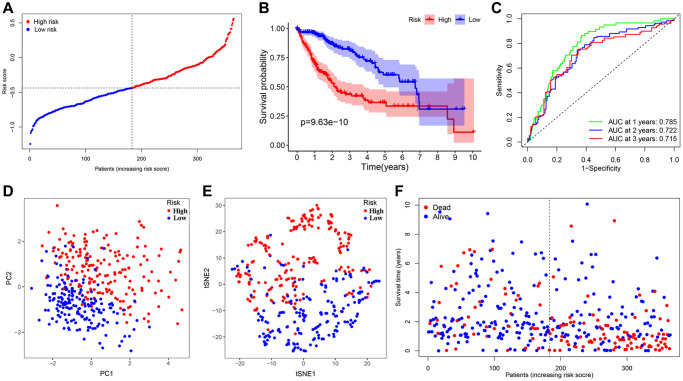
**Establishment and prognostic analysis of a 7-gene signature in the TCGA cohort.** (**A**) The distribution and median value of the risk scores in the TCGA cohort. (**B**) Kaplan-Meier curves for the difference in OS of HCC patients between the high-risk group and low-risk group in the TCGA cohort. (**C**) The AUC of time-dependent ROC curves verified the prognostic performance of the 7-gene signature in the TCGA cohort. (**D**) The PCA plot of the TCGA cohort. (**E**) The t-SNE analysis of the TCGA cohort. (**F**) The distributions of OS status, OS and risk score in the TCGA cohort.

We conducted the least absolute shrinkage and selection operator (LASSO) regression to filter suitable genes from the above expression of the 16 genes to establish a prognostic signature. A 7-gene signature was identified on the basis of the optimal value of λ (Supplementary Figure 1A, 1B). We calculated the risk score as shown below: risk score = (0.123128 × expression of ANGPT1) + (–0.190501 × expression of ENG) + (0.282949 × expression of PDCD10) + (0.122334 × expression of PGF) + (–0.033803 × expression of COL18A1) + (0.016683 × expression of ITGAV) + (–0.066838 × expression of PON1). In terms of the median risk score, 365 HCC patients were dichotomized into a high-risk group (*n* = 182) and a low-risk group (*n* = 183) (Figure 3A). A Kaplan-Meier curve was created to indicate that patients in the low-risk group had a better OS probability than those in the high-risk group (*P* < 0.001) (Figure 3B). The OS rates at 1-, 2-, 3-year for the high- risk group were 69.7%, 53.0%, and 42.2%, whereas the corresponding rates for another group were 95.4%, 85.7%, and 80.9%, respectively. Other similar studies focused on the analysis of disease-specific survival (DSS), progression-free survival (PFS), and disease-free survival (DFS) of these HCC patients, whose outcomes of survival status and Kaplan-Meier curves were highly consistent with the results of OS (Supplementary Figure 2A–2C). To estimate its predictive performance, we displayed the time-dependent receiver operating characteristic (ROC) curve, with the area under the curve (AUC) reaching 0.785, 0.722 and 0.715 in the 1 year, 2 years, and 3 years, respectively, indicating that the prognostic signature exhibited an outstanding specificity and sensitivity (Figure 3C). To study this signature in-depth, we divided the patients into subgroups based on their clinical characteristics (age <=65 and >65, female and male, stage I and II and III and IV, grade G1 & 2 and 3 & 4), and compared the divergences of OS between the two groups in each subgroup. The Kaplan-Meier curves of OS in each subgroup had the same trends as the OS of all samples, suggesting that the signature could be applied to different clinically specific populations (Supplementary Figure 3A–3H).

**Figure 4:**
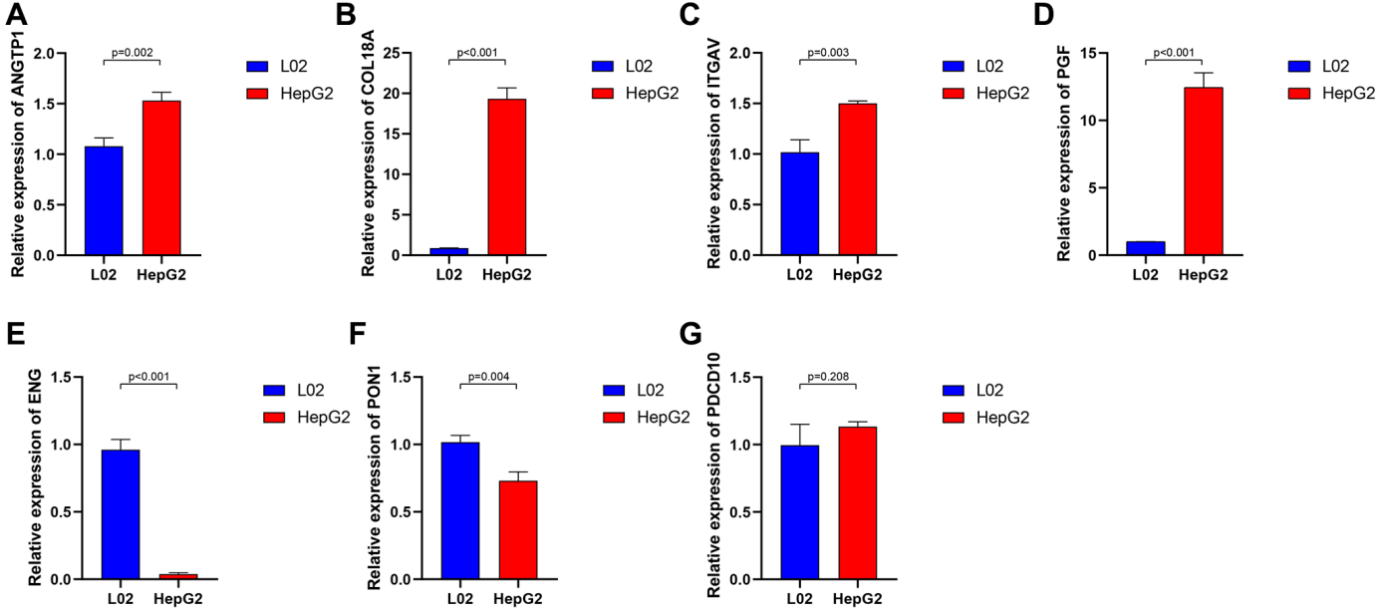
**The relative expression of 7 angiogenesis-related genes between normal liver cell lines and hepatocarcinoma cell lines.** ANGTP1 (**A**), COL18A (**B**), ITGAV (**C**), PGF (**D**) were relatively highly expressed in hepatocarcinoma cell lines, while ENG (**E**), PON1 (**F**) had higher expression in normal liver cell lines. (**G**) There is no significant difference in the expression of PDCD10 in the two cell lines.

**Figure 5:**
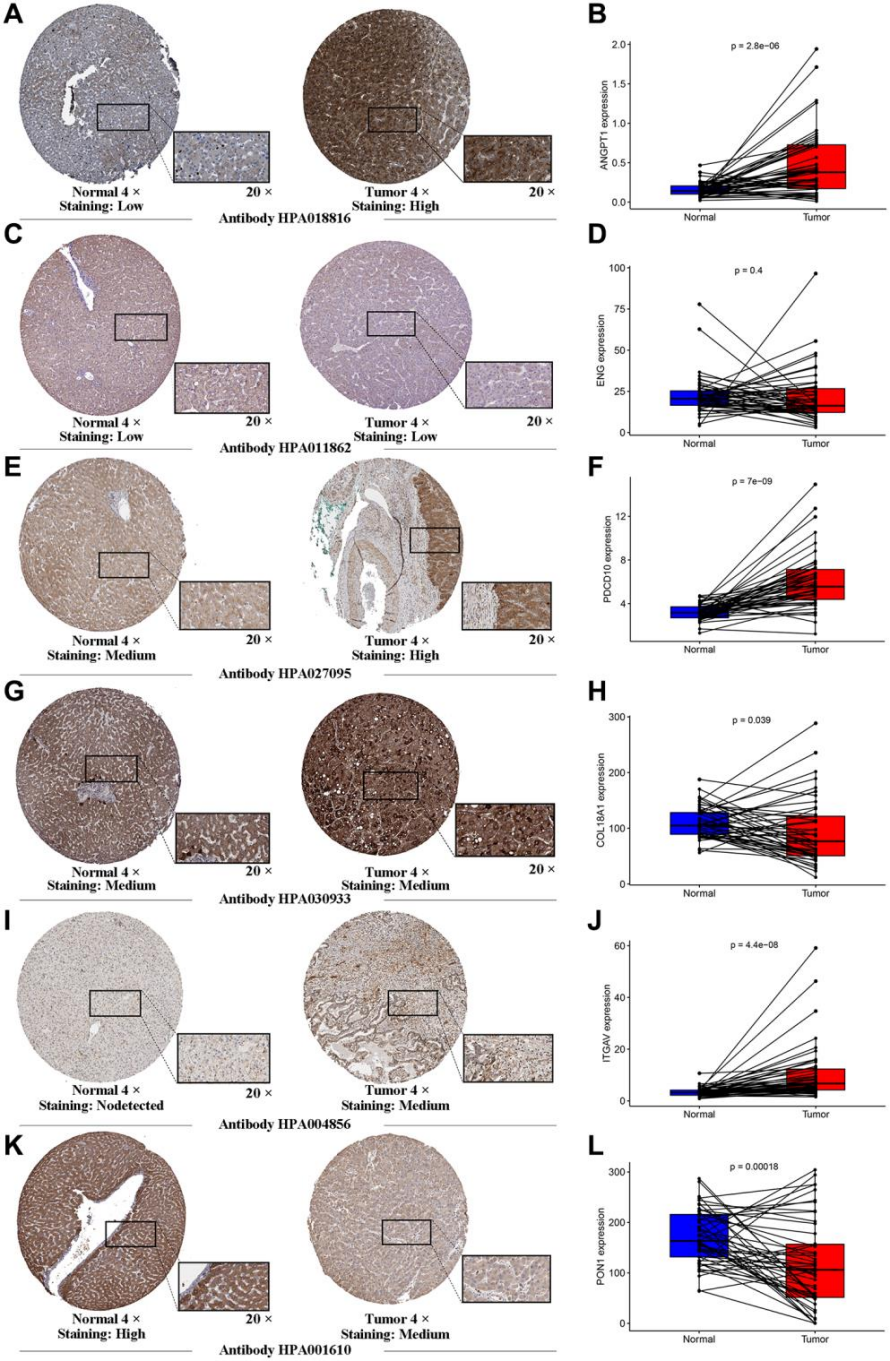
**Human Protein Atlas immunohistochemistry of normal sample and tumor sample.** The expression levels of ANGTP1 (**A**, **B**), ENG (**C**, **D**), PDCD10 (**E**, **F**), COL18A (**G**, **H**), ITGAV (**I**, **J**) and PON1 (**K**, **L**) in tumor and normal tissues were validated in the TCGA cohort, using the paired expression of the same individual normal tissue and tumor tissue.

To further investigate the genes involved in the signature, we examined a human liver cell line (L02) and a hepatoma cell line (HepG2) for the expression of these genes through qRT-PCR. A series of specific primers of each gene were shown in Table 2. The relative expression of each gene between the L02 cell line and HepG2 cell line confirmed by qRT-PCR was not completely consistent with the corresponding RNA-sequence from the TCGA cohort, in light of the high heterogeneity of HCC. The expression of ANGTP1, COL18A, ITGAV, PGF in HepG2 cells were higher than those of L02 (Figure 4A–4D), while ENG and PON1 were highly expressed in hepatocytes (Figure 4E–4F). There was no difference in PDCD10 expression in both cell lines (Figure 4G). We further observed the immunohistochemistry of these 7 proteins in normal and pathological sections through the Human Protein Atlas (HPA) database, which lacked PGF data (Supplementary Figure 4). The comparison results were highly consistent with paired transcriptome sequencing differences (Figure 5A–5L). Survival analyses, on the basis of the optimal cut-off value of each gene expression, revealed that the differential expression of these genes was relevant to the prognosis of HCC patients. Particularly, the relatively high expression of ANGPT1, ITGAV, PDCD10, and PGF were in association with poor prognosis, whereas the higher expression of COL18A1, ENG, and PON1 had relation to a better prognosis, which was completely consistent with the sign of the corresponding gene coefficient in the signature (Supplementary Figure 5A–5G). There was an extremely obvious difference in survival analysis between various risk groups divided by the optimal cut-off configuration as well (Supplementary Figure 5H).

**Table 2:** Sequences of qRT-PCR primer.

**Gene symbol**	**Forward primer sequence**	**Reverse primer sequence**
GAPDH	GGAGCGAGATCCCTCCAAAAT	GGCTGTTGTCATACTTCTCATGG
ANGPT1	CCTGATCTTACACGGTGCTGATT	GTCCCGCAGTATAGAACATTCCA
PDCD10	GCCCCTCTATGCAGTCATGTA	AGCCTTGATGAAAGCGGCTC
ITGAV	ATCTGTGAGGTCGAAACAGGA	TGGAGCATACTCAACAGTCTTTG
ENG	GACCCTGGTACTAAAGAAAGAGC	GAGAGGCTGTCCATGTTGAG
PGF	CATGTTCAGCCCATCCTGTGTCTC	CACCTTTCCGGCTTCATCTTCTCC
COL18A1	CGGGATGAACGGATTGAAAGGAGAG	CCAACTGAAGAAAGTCAAACGGAAACTG
PON1	GGTGAACCATCCAGATGCCAAGTC	TAGTAGACAACATACGACCACGCTAAAC

Meanwhile, Principal component analysis (PCA) and t-distributed stochastic neighbor embedding (t-SNE) analysis demonstrated that HCC patients in various risk groups were distributed in different directions (Figure 3D, 3E). Patients belonging to the latter had lower mortality as well as better prognosis; with the risk score increasing, the probability of death elevated obviously and the OS was significantly shortened (Figure 3F).

### Validation of the 7-gene signature in the ICGC dataset

**Figure 6:**
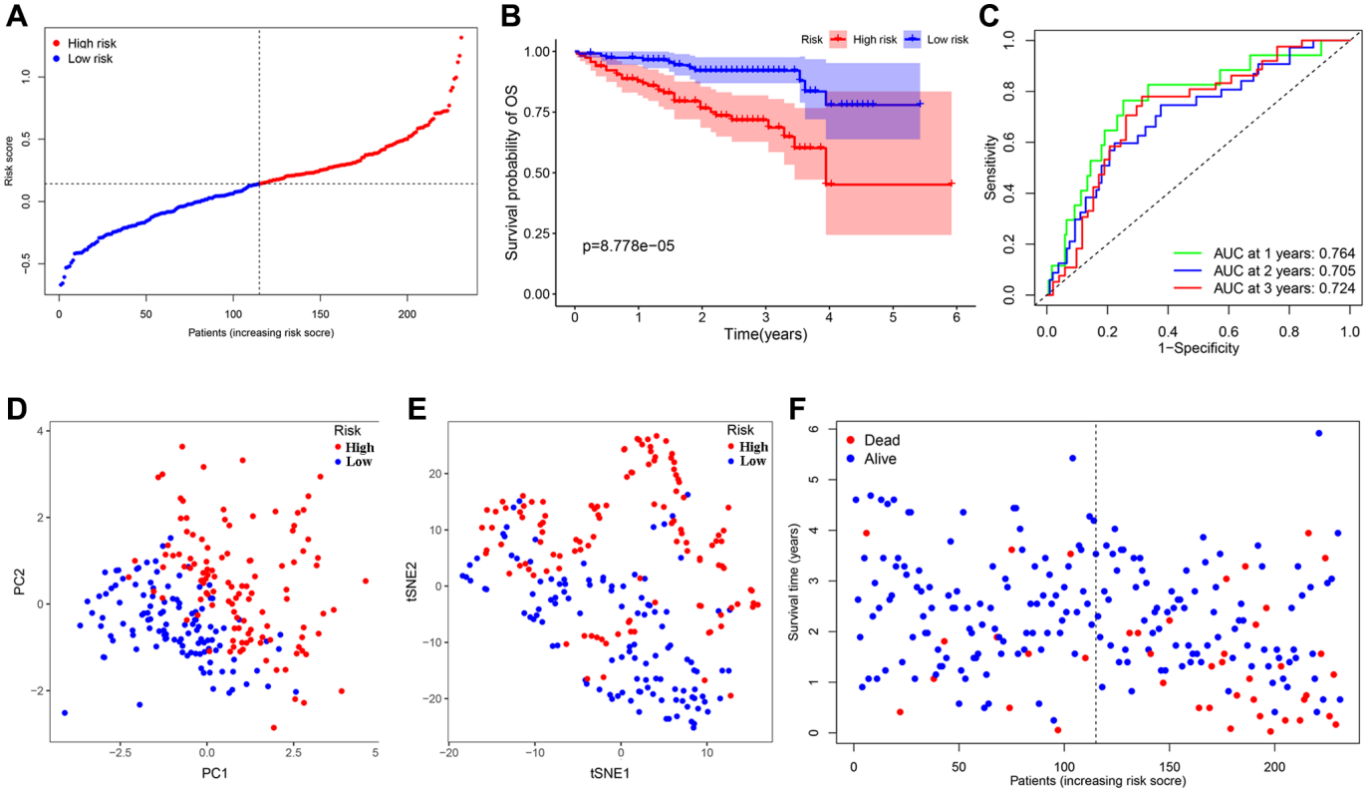
**Validation of the 7-gene signature in the ICGC cohort.** (**A**) The distribution and median value of the risk scores in the ICGC cohort. (**B**) Kaplan-Meier curves for the difference in OS of HCC patients between the high-risk group and low-risk group in the ICGC cohort. (**C**) The AUC of time-dependent ROC curves verified the prognostic performance of the 7-gene signature in the ICGC cohort. (**D**) The PCA plot of the TCGA cohort. (**E**) The t-SNE analysis of the ICGC cohort. (**F**) The distributions of OS status, OS and risk score in the ICGC cohort.

The HCC patients in the ICGC dataset were organized into a high-risk group (*n* = 115) and a low-risk group (*n* = 116) in the light of the median value, calculated with the above-mentioned model formula, to estimate the robustness of the signature what has been established (Figure 6A). Kaplan-Meier analysis suggested that there was a remarkable difference in the survival probability of the two groups (*P* < 0.001) (Figure 6B). The AUC of the 7-gene signature for 1-, 2-, 3-year OS were 0.764, 0.705, and 0.724 respectively (Figure 6C). Similarly, both t-SNE and PCA analysis illustrated that the distribution of these samples in the two risk groups presented discrete directions, which corroborated the results of the TCGA cohort (Figure 6D, 6E). Patients in the high-risk group were confronted with a shorter survival time and more deaths compared with their low-risk counterparts (Figure 6F). Apart from ANGPT1, ITGAV, and PGF, other genes and risk scores were fully related to OS and consistent with the trend of the TCGA outcomes (Supplementary Figure 6A–6H). We divided these HCC patients into different subgroups according to their clinical characteristics and performed survival analysis. And the signature was also applicable to the population with different clinical characteristics in the ICGC dataset (Supplementary Figure 7A–7F).

### Independent prognostic value of the 7-gene signature

After removing samples with incomplete clinical information, the further analysis involved several clinical characteristics of 240 HCC patients, containing age, gender, grade, and stage. We applied univariate and multivariate Cox regression analysis to assess the effectiveness of independent prognostic predictions of risk scores and other clinical characteristics. Univariate analysis indicated that risk score was considerably correlated with the OS probability in the TCGA dataset (HR = 6.160, 95% CI = 3.568–10.633, *P* < 0.001) and the ICGC dataset (HR = 5.953, 95% CI = 2.774–12.775, *P* < 0.001). The risk score was still proved to be an independent predictor for OS in the TCGA dataset (HR = 5.430, 95% CI = 2.998–9.833, *P* < 0.001) and the ICGC dataset (HR = 4.376, 95% CI = 1.987–9.637, *P* < 0.001), after correction for other confounding factors through the multivariate Cox regression analysis. Besides, stage was also an independent prognostic factor for predicting OS (TCGA dataset: HR = 1.996, 95% CI = 1.361–2.927, *P* < 0.001; ICGC dataset: HR = 2.559, 95% CI = 1.330–4.925, *P* = 0.005). The detailed information of the independent prognostic analyses was presented in Table 3, 4.

**Table 3:** Univariate and multivariate analyses of OS in the TCGA cohort.

**Factors**	**Univariate**		**Multivariate**	
	**HR (95%CI)**	***P*-value**	**HR (95% CI)**	***P*-value**
**Age (year) (≤65, >65)**	1.222 (0.839–1.780)	0.295	–	–
**Gender (female, male)**	0.776 (0.531–1.132)	0.188	–	–
**Grade (G1 & G2, G3 & G4)**	1.141 (0.784–1.661)	0.490	–	–
**Stage (TNM I & II, III & IV)**	2.500 (1.721–3.632)	<0.001	1.996 (1.361–2.927)	<0.001
**Risk Score**	6.160 (3.568–10.633)	<0.001	5.430 (2.998–9.833)	<0.001

**Table 4:** Univariate and multivariate analyses of OS in the ICGC cohort.

**Factors**	**Univariate**		**Multivariate**	
	**HR (95% CI)**	***P*-value**	**HR (95% CI)**	***P*-value**
**Age (year) (≤65, >65)**	1.304 (0.690–2.462)	0.413	–	–
**Gender (female, male)**	0.502 (0.268–0.940)	0.031	0.495 (0.253–0.969)	0.040
**Stage (TNM I & II, III & IV)**	2.492 (1.351–4.599)	0.003	2.559 (1.330–4.925)	0.005
**Risk Score**	5.953 (2.774–12.775)	<0.001	4.376 (1.987–9.637)	<0.001

**Figure 7:**
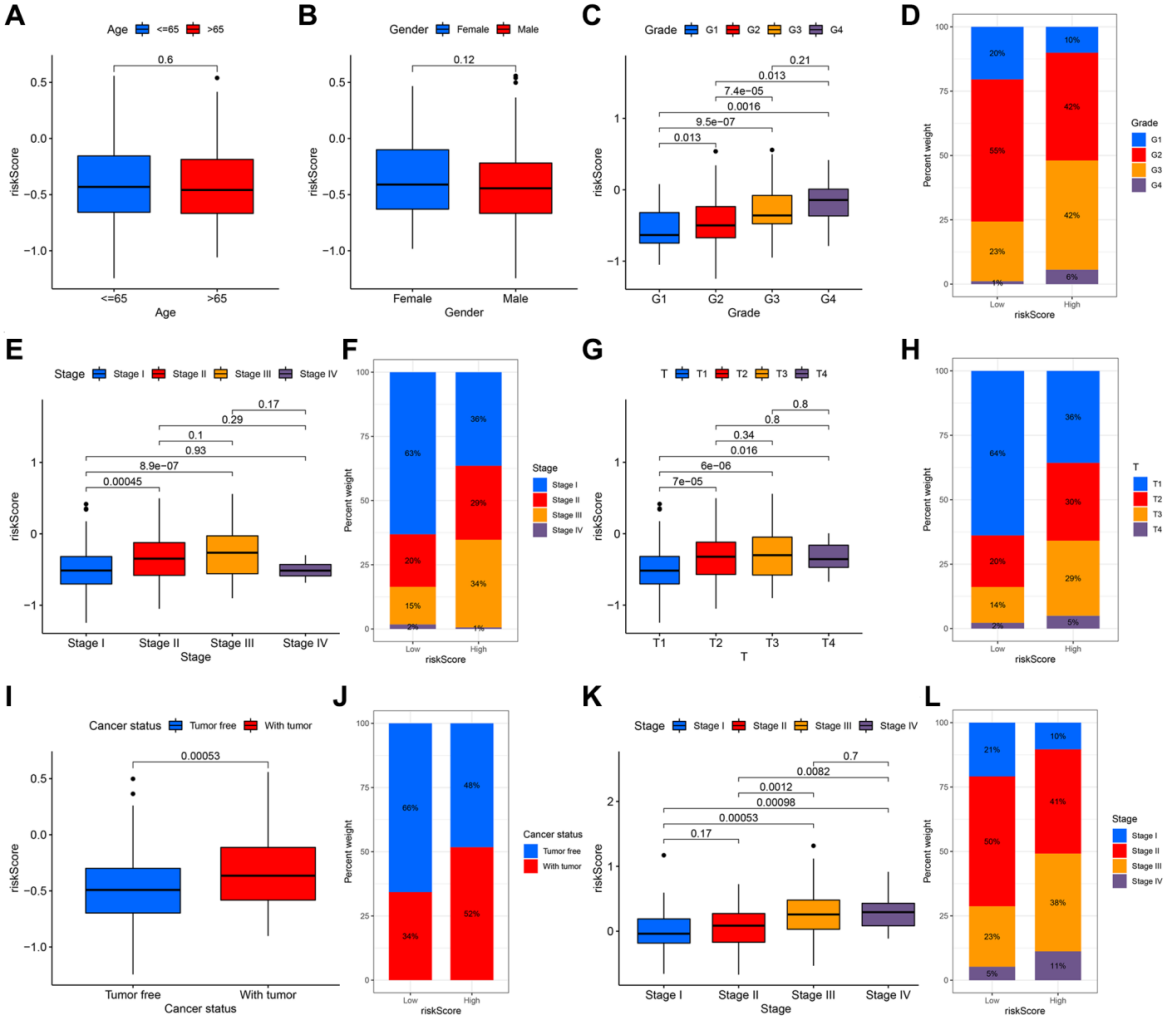
**The relationship between the signature and clinical characteristics of HCC patients.** There was no difference in risk scores for patients of different ages (**A**) and genders (**B**). With the increase of grade (**C**, **D**), stage (**E**, **F**) and T classification (**G**, **H**), the risk had an upward trend. There was a significant difference in the with tumor and tumor free patients (**I**, **J**). The stage difference could be verified in the ICGC cohort (**K**, **L**).

To illustrate the clinical application value of this signature, we compared the differences in risk scores for various clinical characteristics from the TCGA cohort. There were no remarkable differences in risk scores in patients of various ages and genders, indicating that the two have no additional contribution to the prognostic risk in this model (Figure 7A, 7B). It was worth noting that there are obvious differences in the risk scores of this model in different grades, except for G3 and G4, and as the grade increased step by step, the risk scores also increased (Figure 7C). This signature could effectively distinguish the different grades of HCC, so that could be a potential biomarker for HCC grades. The low-risk group was represented by G2, and the high-risk group corresponded to G3 (Figure 7D). Accompanied by the increase of stage and T classifications, risk scores also had a corresponding increase trend; notably, compared with stage II, stage III and other T classifications, the risk scores of stage I and T1were pronouncedly lower (Figure 7E–7H). The angiogenesis status of various stages in the ICGC cohort also had similar outcomes (Figure 7K, 7L). For tumor status, the risk scores of patients with tumor were higher than that of patients with tumor-free, suggesting that the differences in high and low scores of patients in this signature revealed that the total resection could be performed, which is also closely related to the angiogenesis (Figure 7I, 7J).

### A personalized prognostic prediction model

**Figure 8:**
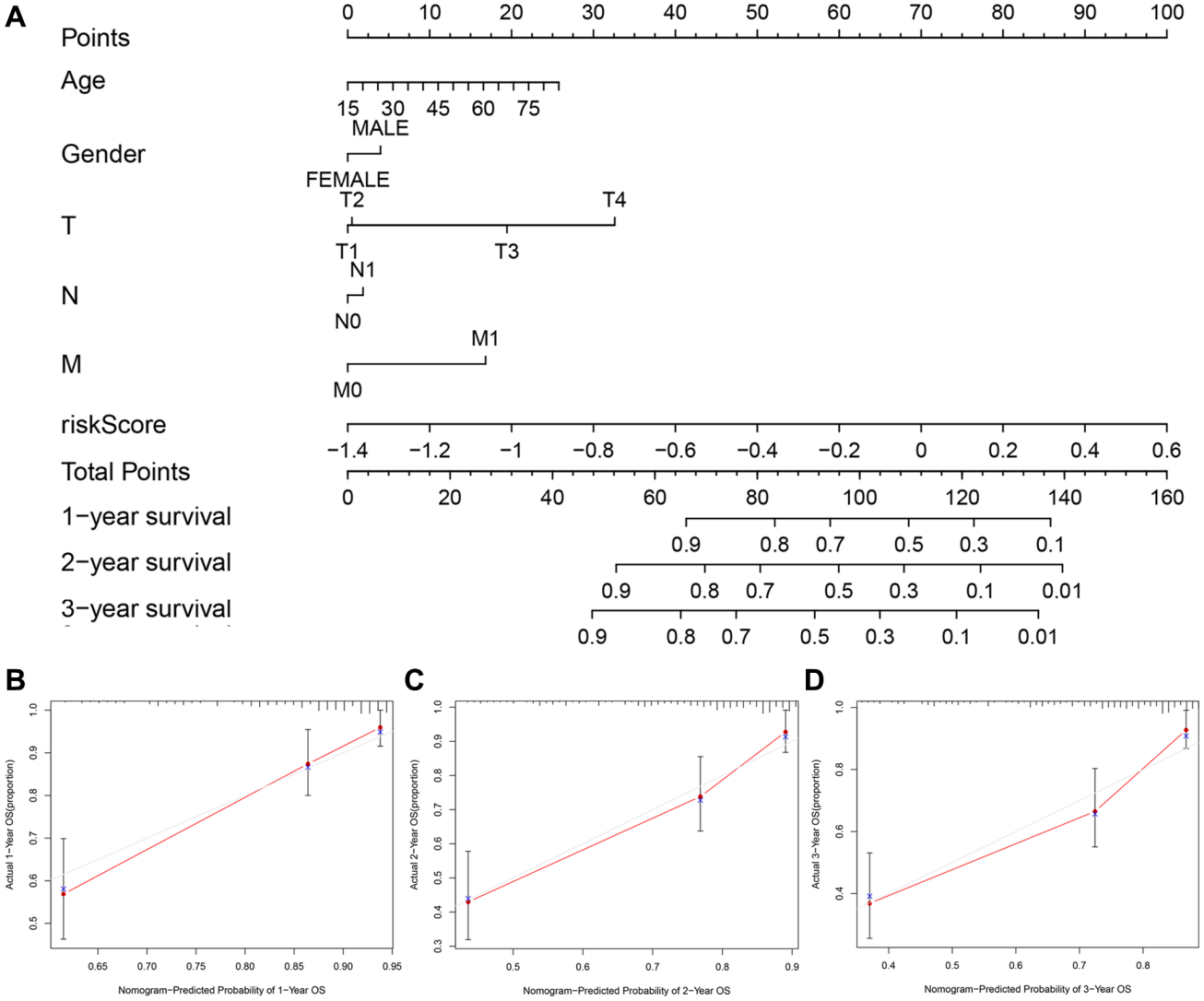
**The nomogram to predict the survival probabilities in the TCGA cohort.** (**A**) The nomogram for predicting OS of HCC patients in the TCGA cohort. The calibration plots for predicting 1-year (**B**), 2-year survival (**C**) and 3-year survival (**D**) in the TCGA dataset.

We applied the nomogram to quantitatively estimate the individual’s actual clinical survival risk by integrating multiple risk factors [19]. We constructed a nomogram by integrating the 7-gene signature, age, gender and TNM classification to forecast the prognosis at 1, 2, and 3 years (Figure 8A). The length of each line represented its degree of influence on the prognosis, and different values corresponded to different results. Especially, the risk score corresponded to the longest line, indicating that it had the strongest predictive ability for OS. The 1-, 2-, and 3-year survival probability of the patient could be judged based on the total points obtained by adding the point of the risk score related to the patient’s prognosis and the points corresponding to the clinical characteristics. The calibration curves implied that the predicted survival probability matched with the actual one well (Figure 8B–8D).

### Functional analysis of the angiogenesis-related 7-gene signature

**Figure 9:**
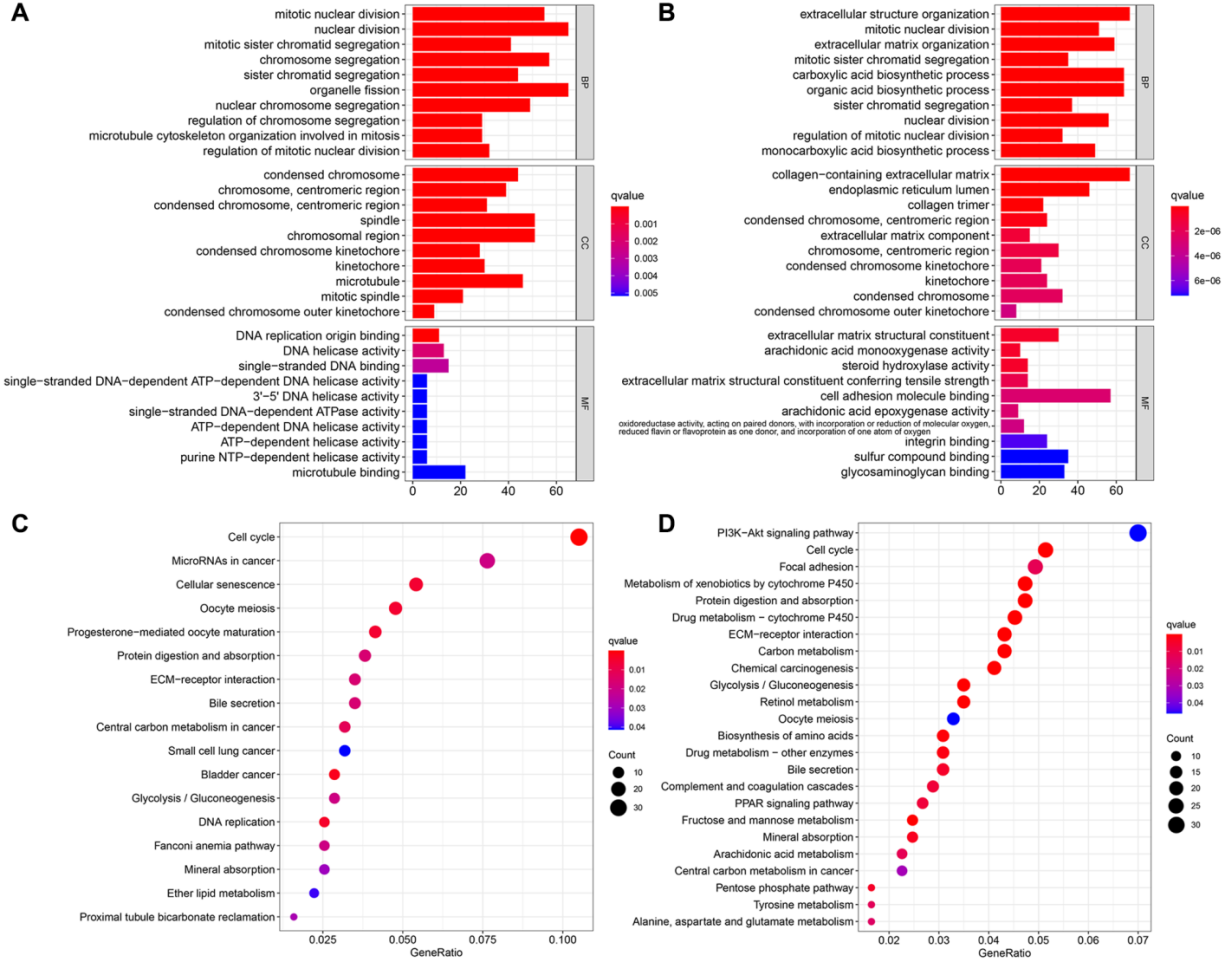
**Functional enrichment analyzes of DEGs.** The most significant or shared GO enrichment and KEGG pathways in the TCGA cohort (**A**, **C**) and the ICGC cohort (**B**, **D**). From top to bottom, the barplot represents the biological process, cellular component, and molecular function, respectively.

We performed GO and KEGG analyses to further clarify the biological pathways and functions relevant to the 7-gene signature through the functional enrichment analysis of DEGs. Conspicuously, DEGs were enriched in several functions concerning cell proliferation and mitosis, as well as nuclear division, which were closed in association to angiogenesis in both datasets (adjusted *P* < 0.05) (Figure 9A, 9B) [20–22]. For biological processes (BP), the major enriched GO terms were nuclear division and organelle fission, and other immune-related pathways, such as neutrophil activation involved in the immune response. The most enriched cellular component (CC) was the spindle and chromosomal region. And it revealed that primary functional categories in molecular function (MF) were microtubule binding and single-stranded DNA binding. Therefore, the application of anti-mitotic drugs such as colchicine may effectively inhibit tumor growth and metastasis. KEGG pathway primarily involved in PI3K-Akt signaling pathway, cell cycle, microRNAs in cancer, ECM-receptor interaction, as well as other cancer-promoting pathways (adjusted *P* < 0.05) (Figure 9C, 9D).

**Figure 10:**
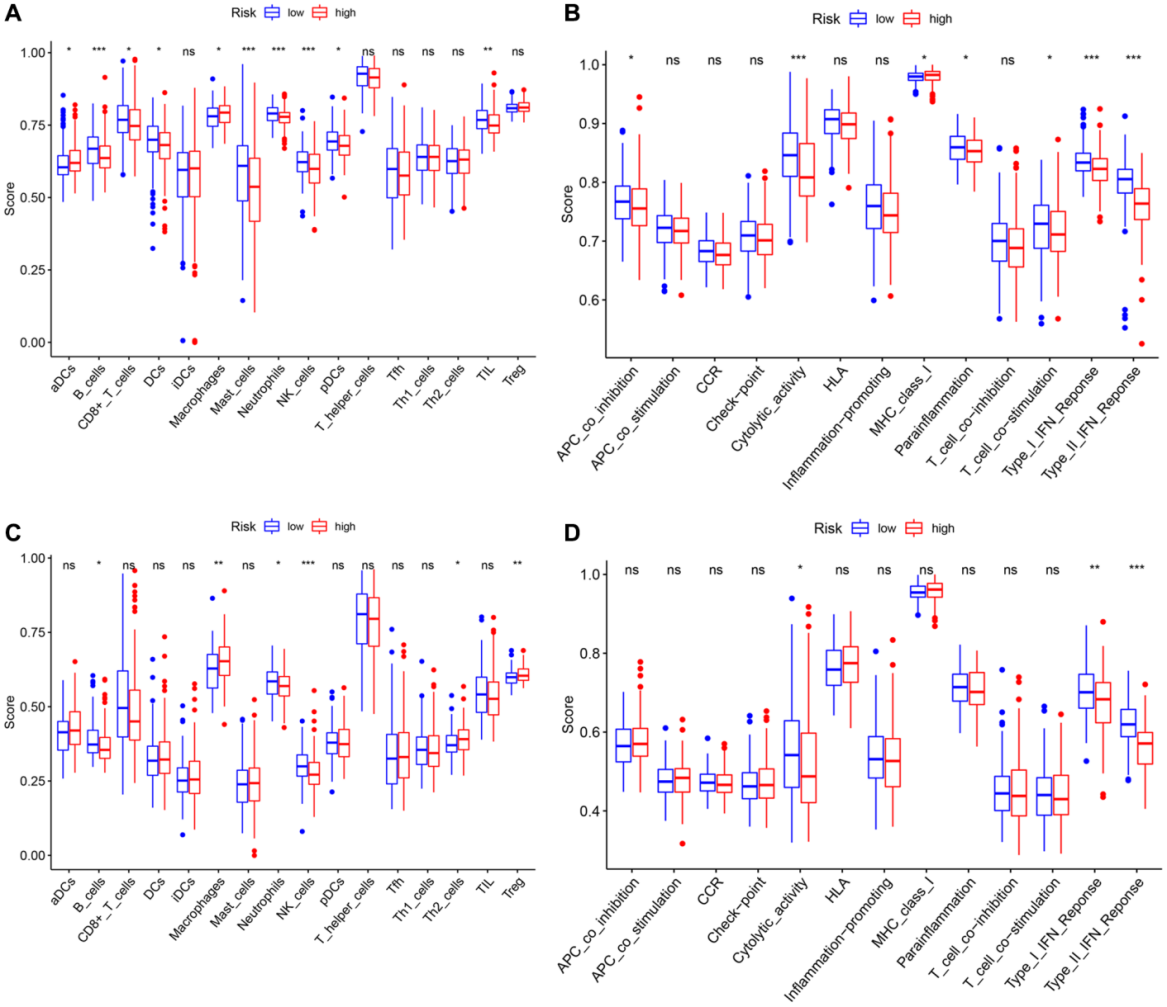
**Comparison of the ssGSEA scores between different risk groups in the TCGA cohort and the ICGC cohort.** The scores of 16 immune cells (**A**, **C**) and 13 immune-related functions (**B**, **D**) are displayed in boxplots. *P* values were showed as: ns: not significant; ^*^P < 0.05; ^**^P < 0.01; ^***^P < 0.001.

Previous studies had shown that tumor anti-angiogenesis therapy went hand in hand with immunotherapy [23–25]. With the normalization of tumor vasculature (or anti-angiogenesis), tissue perfusion was enhanced, and the infiltration of immune effector cells was improved, leading to immunotherapy potentiation; meanwhile, the stimulation of immune cell functions also contributed to normalizing tumor blood vessels. Thus, ssGSEA was utilized to perform enrichment analysis on different immunocyte subsets, related pathways and functions to investigate the relationship between risk scores and immune cells infiltration with and related components. The scores of adaptive immune cells, including CD8+ T cells, B cells, Neutrophils, and TIL, were different between the two groups in the TCGA cohort; notably, the expression values of these cells in the high-risk group were lower than those in the low-risk group (all *P* < 0.05) (Figure 10A). Some molecules and signals related to the antigen presentation process, such as MHC class I, aDCs, DCs, pDCs, and APC co-inhibition, also had gaps between various groups in the TCGA dataset, but it was worth noting that their regulation directions were not consistent (all *P* < 0.05) (Figure 10A, 10B). What’s more, the high-risk group had lower scores of NK cells, cytolytic activity, type I and II IFN response, and mast cells, while the score of macrophages was just the opposite (all *P* < 0.05). Similarly, the differences of B cells, Neutrophils, macrophages, NK cells, cytolytic activity, type I and II IFN response between the two groups were verified in another dataset (all *P* < 0.05) (Figure 10C, 10D).

**Figure 11:**
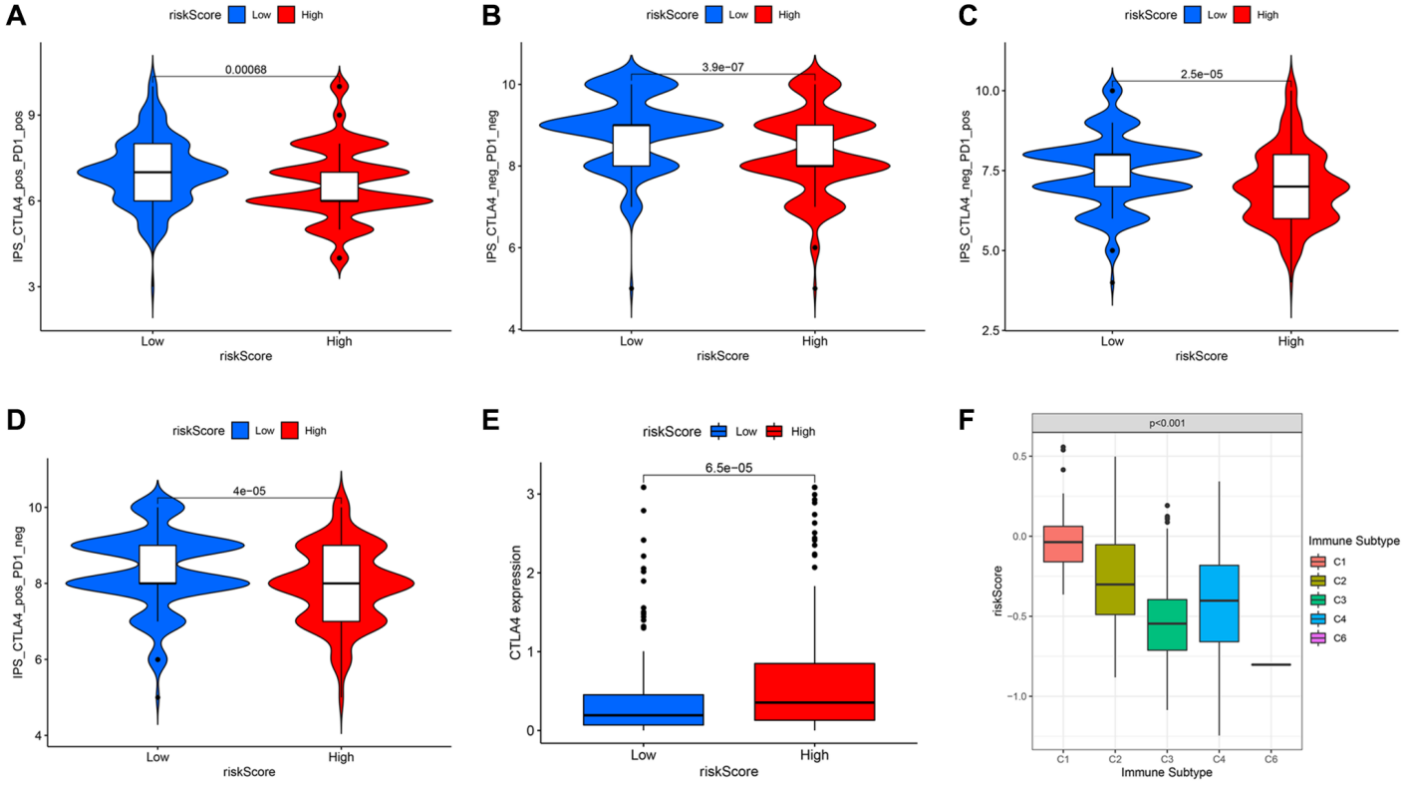
**Comparison of immunophenoscore (IPS) between high and low risk groups under different immune checkpoint states.** In the case of both CTLA4 and PD-1 double-positive (**A**) or double-negative (**B**), or PD-1 positive but CTLA4 negative (**C**), the low-risk group had higher IPS. (**D**) The high-risk group with CTLA4 positive but PD-1 negative had higher IPS. (**E**) The expression level of CTLA4 in different risk groups. (**F**) The risk scores under different immune subtypes.

To further investigate the relation between the model and immunotherapy, the immunophenoscore was utilized to evaluate the distinction in the effect of immunotherapy between the two groups under various immune checkpoint conditions. For HCC patients with both CTLA4 and PD-1 double-positive or double-negative, or PD-1 positive but CTLA4 negative, the IPS of patients in the high-risk group was noticeably lower than that of the low-risk group, that is, under the above-mentioned conditions, patients in the former had a worse effect on immunotherapy (Figure 11A–11C). Yet, a higher IPS was exposed to the patients (with CTLA4 positive but PD-1 negative) in the high-risk group (Figure 11D), resulting from the higher expression of CTLA4 in this group (Figure 11E). Besides, we evaluated the risk scores of the HCC patients corresponding to different immune subtypes (C1: Wound Healing, C2: IFN-gamma Dominant, C3: Inflammatory, C4: Lymphocyte Depleted, C5: Immunologically Quiet, C6: TGF-beta Dominant) (Figure 11F). The immune subtype C1, which is characterized by wound healing, had a remarkably higher risk score than other subtypes. The immune subtype C3 had a relatively low risk score, resulting in a better prognosis, which was completely consistent with existing research conclusions [26].

In general, the immune cell infiltration and corresponding immune response of samples in the low-risk group were dramatically more striking, indicating that relatively normal tumor blood vessels are more likely to be immune cell infiltrated and even beneficial for immunotherapy and combination therapy of anti-angiogenesis therapy and immunotherapy.

## DISCUSSION

As a malignant tumor, HCC often exhibits different degrees of deterioration, metastasis and recurrence under the joint regulation of pro-angiogenesis genes and anti-angiogenesis genes [27]. The high expression of pro-angiogenesis genes has obvious vascular proliferation and vascular abnormalities, including sinusoidal capillarization and arterialization, which promote the metastasis of HCC. On the contrary, the superior expression of anti-angiogenesis genes slows the progression or recurrence of HCC. Therefore, it is of significance to provide prognostic predictions for HCC patients by integrating angiogenesis-related genes as effective and reliable biomarkers. The transcription level of 79 angiogenesis-related genes in tumor tissues of HCC samples has been systematically analyzed, and the relationship between differentially expressed genes and OS probability has also been clarified. A novel potential prognostic signature of 7 angiogenesis-related genes was established and validated by an external dataset. Meanwhile, we performed functional enrichment analyses to identify the pathways relevant to this model and its related pathways of immune infiltration.

Previous studies have shown that pro-angiogenesis genes, such as VEGF, could lead to the progression and metastasis of HCC [28–30]. Also, drugs targeting these pathways have been studied and applied clinically [31]. However, the complex mechanisms of most other angiogenesis-related genes and their combined effects resulted in the unclear relationship between these genes and OS. Of the 79 angiogenesis-related genes selected in this study, 53 genes (67.1%) had a gap in the expression between adjacent normal tissues and tumor tissues of HCC samples, and 25 genes (31.6%) were connected with OS probability through the univariate Cox regression analysis. It is worth noting that in addition to a few star genes, other angiogenesis-related genes had a potential role in the tumorigenesis and progression of HCC, which provided the possibility to establish a potential prognostic signature with multiple angiogenesis-related genes.

This prognostic signature constructed consisted of 7 angiogenesis-related genes, including ANGPT1, ENG, PDCD10, PGF, COL18A1, ITGAV, and PON1. According to the survival analyses of a single gene and the coefficient of each gene in the signature, these genes could be roughly divided into two categories, one of which was related to the poor prognosis of HCC (ANGPT1, ITGAV, PDCD10, PGF), and the other was associated with the suppression of HCC (COL18A1, ENG, PON1). ANGPT1 encodes a secreted glycoprotein, which plays important role in vascular development and angiogenesis, thereby promoting the tumor dedifferentiation and development of HCC [32]. The protein encoded by ENG is a major glycoprotein of the vascular endothelium. However, the relationship between the quantitative endoglin expression and the prognostic effect of HCC is not yet known. Some studies have shown that the expression of endoglin in tumor tissues and the serum level of soluble endoglin are positively related to more advanced clinical stages and poor prognosis [33, 34]. Other studies report that higher expression of ENG microvascular density, cyclooxygenase-2 in endothelial cells of non-tumor tissue, in comparison with tumors, only plays a role in tumorigenesis, but does not promote tumor progression [35, 36]. This view is consistent with the role of ENG in the prognostic signature constructed in this study. The loss of endothelial PDCD10, associated with cell apoptosis, stimulates proliferation and inhibits apoptosis to activate glioma cells and promote tumor growth [37]. There is no relevant research on the expression of PDCD10 in HCC. The expression level of PGF, homologous to VEGF, is relation to the poor prognosis of various cancers, including HCC, colorectal cancer, kidney cancer, and other cancers [38–41]. COL18A1 encodes a potent antiangiogenic protein that can inhibit angiogenesis and HCC tumor growth [42]. The integrin encoded by ITGAV may regulate angiogenesis and promote HCC progression and metastasis. As a pioneer factor, LncRNA AY927503 promoted HCC metastasis by modifying ITGAV transcription [43]. Low expression of PON1 was connected with poor survival in HCC patients [44]. We verified the relative expression of these 7 genes in a normal liver cell line and a hepatocarcinoma cell line by qRT-PCR, and the results were highly consistent with existing researches. Significantly, the sign of the coefficient of each gene in the signature constructed in the current was consistent with the direction of up-regulation or down-regulation in HCC. Whether these genes have a certain impact on the prognosis of HCC patients by affecting the angiogenesis process is still elucidated, because there are few definitive reports on the mechanism of these ones, apart from ANGPT1, COL18A1, and ITGAV.

This angiogenesis-related model is particularly relevant to the clinical characteristics of HCC patients, especially the malignant degree of the tumor itself and the degree of tumor progression. In this study, we found that as the grade and stage of HCC increase, the risk score will also increase accordingly. Regarding the grade of the tumor, except that the risk score of G4 is not different from that of G3, which is only slightly higher than the score of G3, the difference in risk scores between grades is still very obvious. In other words, one reason for the different degrees of malignancy caused by different pathological grades of HCC may be that angiogenesis plays a certain role in this process, that is, the differential expression of angiogenesis-related genes in different pathological grades leads to their invasion. The different ability of metastasis, which in turn leads to different degrees of malignancy. In addition, we can also use this signature as a biomarker for HCC grading to make a preliminary judgment and identification of the malignant degree. Angiogenesis also plays a role in the tumor stage. In the TCGA cohort, the risk scores of stage I differ from the scores of stage II and stage III significantly. There are marked differences between the T1 classification and other T1 classifications, as well. We consider that the difference in the stage is mainly due to the difference in T classification because the main gap between the T1 and other T classifications is whether the vascular invasion occurs. With the increase of the risk, the more abundant angiogenesis-related genes are expressed, which may further cause tumor vascular invasion. Therefore, this model can also be used as an important indicator of T classification.

The tumor susceptibility to angiogenesis has been a hot spot of studies in recent years, while targeted drugs based on these have also been developed and clinically applied. Yet, the potential mechanisms of angiogenesis giving rise to tumor progression and metastasis and related drug resistance are still elusive. Using GO and KEGG analysis to clarify the related pathways of DEGs between the two groups, the results suggested that these genes were mostly focused on the functions and signal pathways of cell proliferation and mitosis. The angiogenesis that we have emphasized earlier that contributes to tumor metastasis may only be a generalized proliferation. As a possible deeper mechanism, these “angiogenesis-related genes” exert critical functions in the process of mitosis, through promoting the interaction and migration of microtubules, which in turn, lead to cell proliferation, not only vascular endothelial cells, but also tumor cells [45]. Moreover, the extensive proliferation of vascular endothelial cells paves the way for subsequent tumor cell proliferation and migration. For these patients with angiogenesis, the application of anti-mitotic drugs such as colchicine may be more beneficial to combat tumor growth and metastasis. The IPS outcomes indicated that immunotherapy has a better effect on patients in the low-risk group. Thus, the signature may become a potential target for immunotherapy. Furthermore, studies in mounting numbers demonstrated that immune cell infiltration in the tumor microenvironment (TME) and anti-angiogenesis or vascular normalization had mutually promoting effects [46–49]. VEGF induces the production of myeloid suppressive cells, regulatory T cells, and other immunosuppressive-related cells, which breaks the immune dynamic balance and develops its inhibitory direction. The anti-angiogenic drugs that antagonize VEGF or disrupt VEGF signal transduction have a positive regulatory effect on the immune effect by promoting tissue perfusion and immune cell infiltration into tumors, thereby enhancing the effect of immunotherapy. Therefore, anti-angiogenesis and normalization of blood vessels could promote the efficacy of immunotherapy by inducing the secretion of adhesion molecules in the cavity of tumor vascular endothelial cells, promoting the infiltration of immune cells into tumor tissues, improving the TME, and ultimately alleviating immunosuppression. Conversely, the increase and activation of effector T cells in the tumor promote the remodeling and normalization of blood vessels and TME. Immunotherapy combined with anti-angiogenesis therapy can transform the battlefield tailored by tumor cells into the other battlefield that is conducive to immune cells attacking tumors by improving the harsh TME, notably, anti-angiogenic drugs may be the magic weapon in this process. The higher fractions of adaptive immune cells in the low-risk group suggested that the relative normalization of tumor blood vessels facilitates the infiltration of immune cells, thereby co-suppress HCC. Therefore, the combined effect of the interaction of immune cell infiltration and anti-angiogenic response in low-risk group patients may explain the corresponding better prognosis.

This study has several shortcomings that need to be solved by follow-up work. Firstly, this 7-gene prognostic signature was established and verified with another public database. It is necessary to provide more prospective clinical data to verify. Then, this model almost represents an optimal prognostic model related to angiogenesis by integrating a large number of angiogenesis-related genes, but some vital genes, like VEGFA, were excluded. Simultaneously, the inherent weakness of supposing only one phenotype to establish a prognostic signature was inevitable, since many other fundamental prognostic genes in HCC may be excluded as well. Besides, the link between the angiogenesis-related genes and immune infiltration, and the underlying mechanism need further experimental exploration.

In short, this study screened 7 angiogenesis-related genes as prognostic biomarkers and established a novel prognostic signature. And it has been validated in association with OS probability in two datasets independently, providing a novel breakthrough in the prognosis of HCC patients. The potential mechanism of angiogenesis-related signatures of HCC in immune infiltration remains relatively little known and deserves further study.

## MATERIALS AND METHODS

### Transcriptome data and clinical data collection

Transcriptome data, containing gene expression, together with clinical characteristics of American HCC samples (*n* = 371) and Japanese HCC samples (*n* = 231) were respectively collected from the TCGA portal (https://portal.gdc.cancer.gov/repository) (up to July 1, 2020) and the ICGC portal (https://dcc.icgc.org/projects/LIRI-JP) (up to August 1, 2020). These Japanese samples were mainly derived from patients with HCC caused by HBV or HCV infection [50]. These extracted RNA-sequence data were normalized with formula log_2_ (*x* + 1) by the “limma” R package. After removing samples with a follow-up time of 0 days and missing clinical characteristics, 365 HCC samples in the TCGA portal, with intact survival status, OS time, age, gender, histological grade, and TNM stage were included. The samples in the ICGC database had complete clinical data. Then, 79 angiogenesis-related genes with a correlation score > 10, provided in Supplementary Table 2, were extracted from the GeneCards database (https://www.genecards.org/).

### Establishment and validation of a potential prognostic angiogenesis-related gene signature

The “limma” R package was used to screen out the DEGs between the tumor tissues and the adjacent normal tissues in the HCC samples in the TCGA data with the false discovery rate (FDR) < 0.05. Then, we screened angiogenesis-related genes that could be utilized to judge the prognosis of HCC patients through univariate cox analysis. The intersection of these two groups of genes was utilized to identify the differentially expressed angiogenesis-related genes with prognostic value in HCC. The STRING database generated an interactive network that overlaps the prognostic DEGs [51]. The interactions with high confidence (0.70) were considered statistically significant. LASSO regression was performed with the “glmnet” R package to establish a prognosis signature to reduce the dimension and enhance its generalization ability [52, 53]. The response variables in the LASSO regression were OS probability and survival status, and the independent variables were the normalized expression matrix of the above candidate prognostic DEGs. Based on tenfold cross-validation following the minimum criteria, we identified the penalty parameter (λ) for the signature. The following computational formula was then established for further analyses:

$$\text{Risk score = }\sum_{i=1}^{n}\left[Expression\;of\;Gene_{i}\times \beta_{i}\right]$$

Where *n* represents the number of finally enrolled genes, β indicates the coefficient of Gene_*i*_ obtained after the LASSO regression.

The patients in both portals were divided into two risk groups for survival analysis in line with the median value of the risk score. And Kaplan–Meier survival analysis was utilized to compare the OS, DFI, PFI, DSS between these two groups. In addition to the above survival analysis, we also utilized the “survminer” R package to identify the optimal cut-off expression to perform survival analysis for each gene and risk score. PCA was carried out to explore whether the difference between the two groups was significantly identified on the basis of unsupervised learning through the “stats” R package. Such dimensionality reduction can extract the portions of the principal components of various groups, so as to clarify whether there were remarkable differences between the two groups. In order to determine the complex structural relationship between features, we performed t-SNE to investigate the distribution of the two groups with the “Rtsne” R package. The time-dependent ROC curves of 1-, 2-, 3-year was implemented to assess the predictive power by the “survivalROC” R package. The paired expression value of angiogenesis-related genes in the signature, along with immunohistochemistry of the expressed proteins were obtained from the HPA database.

### Detection of the expression of each gene in the cell lines by qRT-PCR

We extracted total RNA extracted from the human hepatocarcinoma cell line HepG2 and normal liver cell line L02 using the RNA-Quick Purification Kit (Shanghai Yishan Biotechnology Co., Ltd, China). The A260/A280 absorption (1.9–2.2) was used to evaluate the quality of the extracted total RNA. Then, the HiScript III All-in-one RT SuperMix Perfect for qPCR kit (Vazyme, China) was utilized to reverse transcribe the total RNA as following steps: 50°C for 15 min and 85°C for 5s. The Taq Pro Universal SYBR qPCR Master Mix kit (Vazyme, China) was utilized to perform PCR according to the following reaction procedure: 95°C for 30s, 95°C for 10s with 40 cycles, and 60°C for 30s. The specific primer sequences of GAPDH and these genes were displayed in Table 2. We calculated the relative expression of target genes using the 2^−ΔΔCT^ method.

### Functional enrichment analysis

GO and KEGG analyses were conducted to clarify the enrichment of related functional pathways on the basis of the DEGs (FDR < 0.05, |log2FC| ≥ 1) using the “clusterProfiler” R package. The ssGSEA could be utilized to calculate the infiltrating score of 16 immune cells and the activity of 13 immune-related pathways [54]. Supplementary Table 3 displays the annotated gene set file.

### Development of nomogram

The nomogram can predict the probability of a certain clinical outcome on the basis of the values of multiple variables [55]. To establish a nomogram for the TCGA dataset, we integrated age, gender, TNM classification and risk score with the “survival” and “rms” R package. Then, we plotted calibration curves to estimate the concordance between predicted and actual survival probability. Combining clinical characteristics and risk scores could predict 1-, 2-, 3-year survival rates of HCC patients.

### Statistical analysis

All statistical analyses were performed with R software version 4.0.1 (https://www.R-project.org). Unless otherwise noted, *p* < 0.05 was considered to be statistically significant. We used Chi-square tests to evaluate the differences in proportions, and independent *t*-tests to assess the differences in gene expression levels between adjacent normal tissues and tumor tissues of HCC samples. Mann-Whitney test was utilized to evaluate the ssGSEA scores of pathways and immune cells between different groups for comparison. Kaplan-Meier curves were usually utilized to analyze the survival probability between different groups. Wilcoxon test and Kruskal-Wallis test were performed to compare the differences in risk scores and IPS between different groups. We implemented Cox regression to distinguish independent predictors of OS probability.

## Supplementary Materials

Supplementary Figures

Supplementary Tables 1 and 2

Supplementary Table 3

## References

[1] Bray F, Ferlay J, Soerjomataram I, Siegel RL, Torre LA, Jemal A. Global cancer statistics 2018: GLOBOCAN estimates of incidence and mortality worldwide for 36 cancers in 185 countries. CA Cancer J Clin. 2018; 68:394–424. 10.3322/caac.21492 30207593

[2] Yang JD, Hainaut P, Gores GJ, Amadou A, Plymoth A, Roberts LR. A global view of hepatocellular carcinoma: trends, risk, prevention and management. Nat Rev Gastroenterol Hepatol. 2019; 16:589–604. 10.1038/s41575-019-0186-y 31439937 PMC6813818

[3] Forner A, Reig M, Bruix J. Hepatocellular carcinoma. Lancet. 2018; 391:1301–14. 10.1016/S0140-6736(18)30010-2 29307467

[4] Torrecilla S, Sia D, Harrington AN, Zhang Z, Cabellos L, Cornella H, Moeini A, Camprecios G, Leow WQ, Fiel MI, Hao K, Bassaganyas L, Mahajan M, et al. Trunk mutational events present minimal intra- and inter-tumoral heterogeneity in hepatocellular carcinoma. J Hepatol. 2017; 67:1222–31. 10.1016/j.jhep.2017.08.013 28843658

[5] Lin DC, Mayakonda A, Dinh HQ, Huang P, Lin L, Liu X, Ding LW, Wang J, Berman BP, Song EW, Yin D, Koeffler HP. Genomic and Epigenomic Heterogeneity of Hepatocellular Carcinoma. Cancer Res. 2017; 77:2255–65. 10.1158/0008-5472.CAN-16-2822 28302680 PMC5413372

[6] Bikfalvi A. History and conceptual developments in vascular biology and angiogenesis research: a personal view. Angiogenesis. 2017; 20:463–78. 10.1007/s10456-017-9569-2 28741165

[7] Carmeliet P, Jain RK. Principles and mechanisms of vessel normalization for cancer and other angiogenic diseases. Nat Rev Drug Discov. 2011; 10:417–27. 10.1038/nrd3455 21629292

[8] Viallard C, Larrivée B. Tumor angiogenesis and vascular normalization: alternative therapeutic targets. Angiogenesis. 2017; 20:409–26. 10.1007/s10456-017-9562-9 28660302

[9] De Palma M, Biziato D, Petrova TV. Microenvironmental regulation of tumour angiogenesis. Nat Rev Cancer. 2017; 17:457–74. 10.1038/nrc.2017.51 28706266

[10] Sajib S, Zahra FT, Lionakis MS, German NA, Mikelis CM. Mechanisms of angiogenesis in microbe-regulated inflammatory and neoplastic conditions. Angiogenesis. 2018; 21:1–14. 10.1007/s10456-017-9583-4 29110215

[11] Zhu AX, Park JO, Ryoo BY, Yen CJ, Poon R, Pastorelli D, Blanc JF, Chung HC, Baron AD, Pfiffer TE, Okusaka T, Kubackova K, Trojan J, et al, and REACH Trial Investigators. Ramucirumab versus placebo as second-line treatment in patients with advanced hepatocellular carcinoma following first-line therapy with sorafenib (REACH): a randomised, double-blind, multicentre, phase 3 trial. Lancet Oncol. 2015; 16:859–70. 10.1016/S1470-2045(15)00050-9 26095784

[12] European Association for the Study of the Liver. EASL Clinical Practice Guidelines: Management of hepatocellular carcinoma. J Hepatol. 2018; 69:182–236. 10.1016/j.jhep.2018.03.019 29628281

[13] Keating GM. Sorafenib: A Review in Hepatocellular Carcinoma. Target Oncol. 2017; 12:243–53. 10.1007/s11523-017-0484-7 28299600

[14] Liepelt A, Tacke F. Stromal cell-derived factor-1 (SDF-1) as a target in liver diseases. Am J Physiol Gastrointest Liver Physiol. 2016; 311:G203–09. 10.1152/ajpgi.00193.2016 27313175

[15] Dong ZR, Sun D, Yang YF, Zhou W, Wu R, Wang XW, Shi K, Yan YC, Yan LJ, Yao CY, Chen ZQ, Zhi XT, Li T. TMPRSS4 Drives Angiogenesis in Hepatocellular Carcinoma by Promoting HB-EGF Expression and Proteolytic Cleavage. Hepatology. 2020; 72:923–39. 10.1002/hep.31076 31867749

[16] Wen Y, Zhou X, Lu M, He M, Tian Y, Liu L, Wang M, Tan W, Deng Y, Yang X, Mayer MP, Zou F, Chen X. Bclaf1 promotes angiogenesis by regulating HIF-1α transcription in hepatocellular carcinoma. Oncogene. 2019; 38:1845–59. 10.1038/s41388-018-0552-1 30367150 PMC6462866

[17] Yan Q, Jiang L, Liu M, Yu D, Zhang Y, Li Y, Fang S, Li Y, Zhu YH, Yuan YF, Guan XY. *ANGPTL1* Interacts with Integrin α1β1 to Suppress HCC Angiogenesis and Metastasis by Inhibiting JAK2/STAT3 Signaling. Cancer Res. 2017; 77:5831–45. 10.1158/0008-5472.CAN-17-0579 28904065

[18] Gu F, Yuan S, Liu L, Zhu P, Yang Y, Pan Z, Zhou W. EYA4 serves as a prognostic biomarker in hepatocellular carcinoma and suppresses tumour angiogenesis and metastasis. J Cell Mol Med. 2019; 23:4208–16. 10.1111/jcmm.14309 30957411 PMC6533515

[19] Iasonos A, Schrag D, Raj GV, Panageas KS. How to build and interpret a nomogram for cancer prognosis. J Clin Oncol. 2008; 26:1364–70. 10.1200/JCO.2007.12.9791 18323559

[20] Protopsaltis NJ, Liang W, Nudleman E, Ferrara N. Interleukin-22 promotes tumor angiogenesis. Angiogenesis. 2019; 22:311–23. 10.1007/s10456-018-9658-x 30539314

[21] Zhou Y, Shan S, Li ZB, Xin LJ, Pan DS, Yang QJ, Liu YP, Yue XP, Liu XR, Gao JZ, Zhang JW, Ning ZQ, Lu XP. CS2164, a novel multi-target inhibitor against tumor angiogenesis, mitosis and chronic inflammation with anti-tumor potency. Cancer Sci. 2017; 108:469–77. 10.1111/cas.13141 28004478 PMC5378272

[22] Liu JJ, Higgins B, Ju G, Kolinsky K, Luk KC, Packman K, Pizzolato G, Ren Y, Thakkar K, Tovar C, Zhang Z, Wovkulich PM. Discovery of a highly potent, orally active mitosis/angiogenesis inhibitor r1530 for the treatment of solid tumors. ACS Med Chem Lett. 2013; 4:259–63. 10.1021/ml300351e 24900658 PMC4027509

[23] Tian L, Goldstein A, Wang H, Ching Lo H, Sun Kim I, Welte T, Sheng K, Dobrolecki LE, Zhang X, Putluri N, Phung TL, Mani SA, Stossi F, et al. Mutual regulation of tumour vessel normalization and immunostimulatory reprogramming. Nature. 2017; 544:250–54. 10.1038/nature21724 28371798 PMC5788037

[24] Albini A, Bruno A, Noonan DM, Mortara L. Contribution to Tumor Angiogenesis From Innate Immune Cells Within the Tumor Microenvironment: Implications for Immunotherapy. Front Immunol. 2018; 9:527. 10.3389/fimmu.2018.00527 29675018 PMC5895776

[25] Huang Y, Kim BYS, Chan CK, Hahn SM, Weissman IL, Jiang W. Improving immune-vascular crosstalk for cancer immunotherapy. Nat Rev Immunol. 2018; 18:195–203. 10.1038/nri.2017.145 29332937 PMC5922422

[26] Thorsson V, Gibbs DL, Brown SD, Wolf D, Bortone DS, Ou Yang TH, Porta-Pardo E, Gao GF, Plaisier CL, Eddy JA, Ziv E, Culhane AC, Paull EO, et al, and Cancer Genome Atlas Research Network. The Immune Landscape of Cancer. Immunity. 2018; 48:812–830.e14. 10.1016/j.immuni.2018.03.023 29628290 PMC5982584

[27] Semela D, Dufour JF. Angiogenesis and hepatocellular carcinoma. J Hepatol. 2004; 41:864–80. 10.1016/j.jhep.2004.09.006 15519663

[28] Morse MA, Sun W, Kim R, He AR, Abada PB, Mynderse M, Finn RS. The Role of Angiogenesis in Hepatocellular Carcinoma. Clin Cancer Res. 2019; 25:912–20. 10.1158/1078-0432.CCR-18-1254 30274981

[29] Campagnolo L, Telesca C, Massimiani M, Stuhlmann H, Angelico M, Lenci I, Manzia TM, Tariciotti L, Lehmann G, Baiocchi L. Different expression of VEGF and EGFL7 in human hepatocellular carcinoma. Dig Liver Dis. 2016; 48:76–80. 10.1016/j.dld.2015.09.019 26542361

[30] Choi JW, Cho HR, Lee K, Jung JK, Kim HC. Modified Rat Hepatocellular Carcinoma Models Overexpressing Vascular Endothelial Growth Factor. J Vasc Interv Radiol. 2018; 29:1604–12. 10.1016/j.jvir.2018.07.005 30293733

[31] Hilmi M, Neuzillet C, Calderaro J, Lafdil F, Pawlotsky JM, Rousseau B. Angiogenesis and immune checkpoint inhibitors as therapies for hepatocellular carcinoma: current knowledge and future research directions. J Immunother Cancer. 2019; 7:333. 10.1186/s40425-019-0824-5 31783782 PMC6884868

[32] Torimura T, Ueno T, Kin M, Harada R, Taniguchi E, Nakamura T, Sakata R, Hashimoto O, Sakamoto M, Kumashiro R, Sata M, Nakashima O, Yano H, Kojiro M. Overexpression of angiopoietin-1 and angiopoietin-2 in hepatocellular carcinoma. J Hepatol. 2004; 40:799–807. 10.1016/j.jhep.2004.01.027 15094228

[33] Yagmur E, Rizk M, Stanzel S, Hellerbrand C, Lammert F, Trautwein C, Wasmuth HE, Gressner AM. Elevation of endoglin (CD105) concentrations in serum of patients with liver cirrhosis and carcinoma. Eur J Gastroenterol Hepatol. 2007; 19:755–61. 10.1097/MEG.0b013e3282202bea 17700260

[34] Yang LY, Lu WQ, Huang GW, Wang W. Correlation between CD105 expression and postoperative recurrence and metastasis of hepatocellular carcinoma. BMC Cancer. 2006; 6:110. 10.1186/1471-2407-6-110 16650286 PMC1475877

[35] Paschoal JP, Bernardo V, Canedo NH, Ribeiro OD, Caroli-Bottino A, Pannain VL. Microvascular density of regenerative nodule to small hepatocellular carcinoma by automated analysis using CD105 and CD34 immunoexpression. BMC Cancer. 2014; 14:72. 10.1186/1471-2407-14-72 24507660 PMC3923987

[36] Ribeiro OD, Canedo NH, Pannain VL. Immunohistochemical angiogenic biomarkers in hepatocellular carcinoma and cirrhosis: correlation with pathological features. Clinics (Sao Paulo). 2016; 71:639–43. 10.6061/clinics/2016(11)04 27982164 PMC5108172

[37] Zhu Y, Zhao K, Prinz A, Keyvani K, Lambertz N, Kreitschmann-Andermahr I, Lei T, Sure U. Loss of endothelial programmed cell death 10 activates glioblastoma cells and promotes tumor growth. Neuro Oncol. 2016; 18:538–48. 10.1093/neuonc/nov155 26254477 PMC4799675

[38] Zhang L, Chen J, Ke Y, Mansel RE, Jiang WG. Expression of Placenta growth factor (PlGF) in non-small cell lung cancer (NSCLC) and the clinical and prognostic significance. World J Surg Oncol. 2005; 3:68. 10.1186/1477-7819-3-68 16223445 PMC1276823

[39] Wei SC, Tsao PN, Yu SC, Shun CT, Tsai-Wu JJ, Wu CH, Su YN, Hsieh FJ, Wong JM. Placenta growth factor expression is correlated with survival of patients with colorectal cancer. Gut. 2005; 54:666–72. 10.1136/gut.2004.050831 15831913 PMC1774482

[40] Chen CN, Hsieh FJ, Cheng YM, Cheng WF, Su YN, Chang KJ, Lee PH. The significance of placenta growth factor in angiogenesis and clinical outcome of human gastric cancer. Cancer Lett. 2004; 213:73–82. 10.1016/j.canlet.2004.05.020 15312686

[41] Parr C, Watkins G, Boulton M, Cai J, Jiang WG. Placenta growth factor is over-expressed and has prognostic value in human breast cancer. Eur J Cancer. 2005; 41:2819–27. 10.1016/j.ejca.2005.07.022 16275058

[42] Coulon S, Heindryckx F, Geerts A, Van Steenkiste C, Colle I, Van Vlierberghe H. Angiogenesis in chronic liver disease and its complications. Liver Int. 2011; 31:146–62. 10.1111/j.1478-3231.2010.02369.x 21073649

[43] Kang CL, Qi B, Cai QQ, Fu LS, Yang Y, Tang C, Zhu P, Chen QW, Pan J, Chen MH, Wu XZ. LncRNA AY promotes hepatocellular carcinoma metastasis by stimulating *ITGAV* transcription. Theranostics. 2019; 9:4421–36. 10.7150/thno.32854 31285770 PMC6599657

[44] Zhang Y, Ying X, Zhao Q, Ma J, Zhang D, He C, Han S. Identification of Protein Expression Changes in Hepatocellular Carcinoma through iTRAQ. Dis Markers. 2020; 2020:2632716. 10.1155/2020/2632716 32076459 PMC7008262

[45] Nieuwenhuis J, Brummelkamp TR. The Tubulin Detyrosination Cycle: Function and Enzymes. Trends Cell Biol. 2019; 29:80–92. 10.1016/j.tcb.2018.08.003 30213517

[46] Ribatti D. Mast cells and macrophages exert beneficial and detrimental effects on tumor progression and angiogenesis. Immunol Lett. 2013; 152:83–88. 10.1016/j.imlet.2013.05.003 23685256

[47] Georganaki M, van Hooren L, Dimberg A. Vascular Targeting to Increase the Efficiency of Immune Checkpoint Blockade in Cancer. Front Immunol. 2018; 9:3081. 10.3389/fimmu.2018.03081 30627131 PMC6309238

[48] Wang Q, Gao J, Di W, Wu X. Anti-angiogenesis therapy overcomes the innate resistance to PD-1/PD-L1 blockade in VEGFA-overexpressed mouse tumor models. Cancer Immunol Immunother. 2020; 69:1781–99. 10.1007/s00262-020-02576-x 32347357 PMC11027722

[49] Fukumura D, Kloepper J, Amoozgar Z, Duda DG, Jain RK. Enhancing cancer immunotherapy using antiangiogenics: opportunities and challenges. Nat Rev Clin Oncol. 2018; 15:325–40. 10.1038/nrclinonc.2018.29 29508855 PMC5921900

[50] Fujimoto A, Furuta M, Totoki Y, Tsunoda T, Kato M, Shiraishi Y, Tanaka H, Taniguchi H, Kawakami Y, Ueno M, Gotoh K, Ariizumi S, Wardell CP, et al. Erratum: Whole-genome mutational landscape and characterization of noncoding and structural mutations in liver cancer. Nat Genet. 2016; 48:700. 10.1038/ng0616-700a 27230686

[51] Szklarczyk D, Gable AL, Lyon D, Junge A, Wyder S, Huerta-Cepas J, Simonovic M, Doncheva NT, Morris JH, Bork P, Jensen LJ, Mering CV. STRING v11: protein-protein association networks with increased coverage, supporting functional discovery in genome-wide experimental datasets. Nucleic Acids Res. 2019; 47:D607–13. 10.1093/nar/gky1131 30476243 PMC6323986

[52] Xiang ZJ, Wang Y, Ramadge PJ. Screening Tests for Lasso Problems. IEEE Trans Pattern Anal Mach Intell. 2017; 39:1008–27. 10.1109/TPAMI.2016.2568185 27187950

[53] Wang J, Fan W, Ye J. Fused Lasso Screening Rules via the Monotonicity of Subdifferentials. IEEE Trans Pattern Anal Mach Intell. 2015; 37:1806–20. 10.1109/TPAMI.2014.2388203 26353128

[54] Rooney MS, Shukla SA, Wu CJ, Getz G, Hacohen N. Molecular and genetic properties of tumors associated with local immune cytolytic activity. Cell. 2015; 160:48–61. 10.1016/j.cell.2014.12.033 25594174 PMC4856474

[55] Park SY. Nomogram: An analogue tool to deliver digital knowledge. J Thorac Cardiovasc Surg. 2018; 155:1793. 10.1016/j.jtcvs.2017.12.107 29370910

