# Supplementary material for: Screening and identification of angiogenesis-related genes as potential novel prognostic biomarkers of hepatocellular carcinoma through bioinformatics analysis
Source: Aging (Albany NY). 2021 Jul 12;13(13):17707–33. doi: 10.18632/aging.203260 (PMC8312452; doi:10.18632/aging.203260)
Supplement: Supplementary Figures [file aging-13-203260-s001.pdf]

SUPPLEMENTARY FIGURES

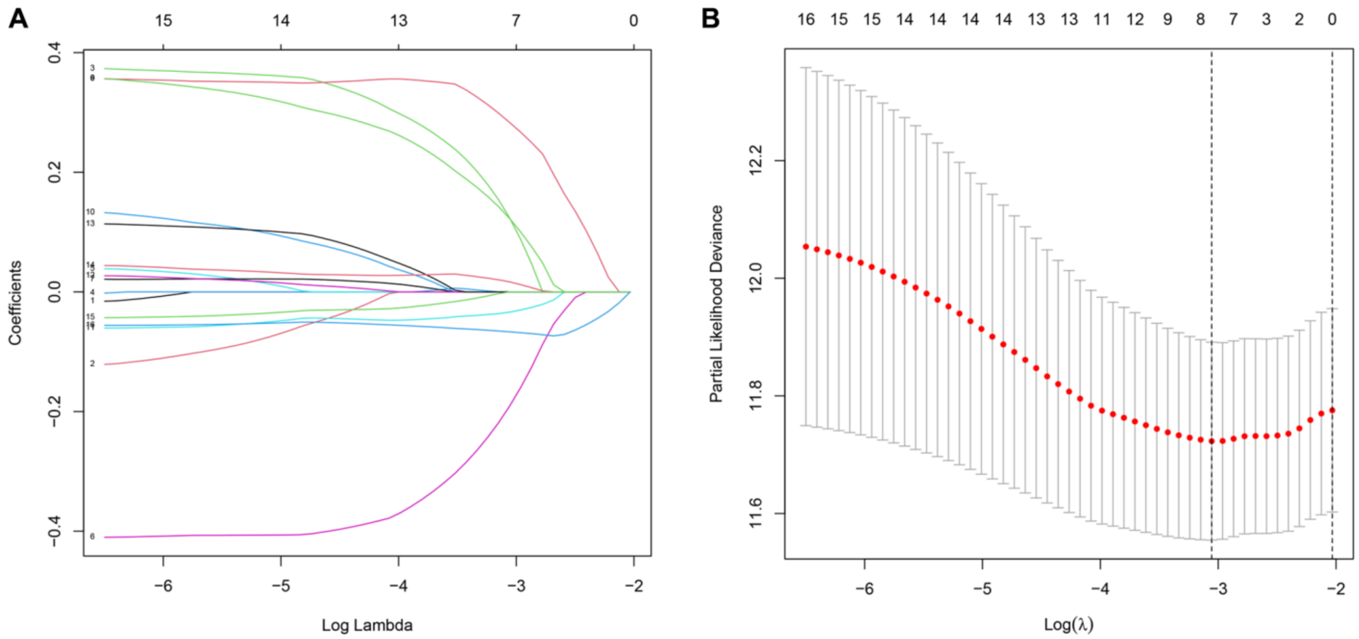

Supplementary Figure 1. Construction of a 7-gene signature model with the LASSO Cox regression analysis in the TCGA cohort. (**A**) Lasso coefficient profiles of the expression of 16 genes in the TCGA cohort. (**B**) A coefficient profile plot was generated against the log (λ) sequence. Selection of the optimal parameter (λ) in the Lasso model.

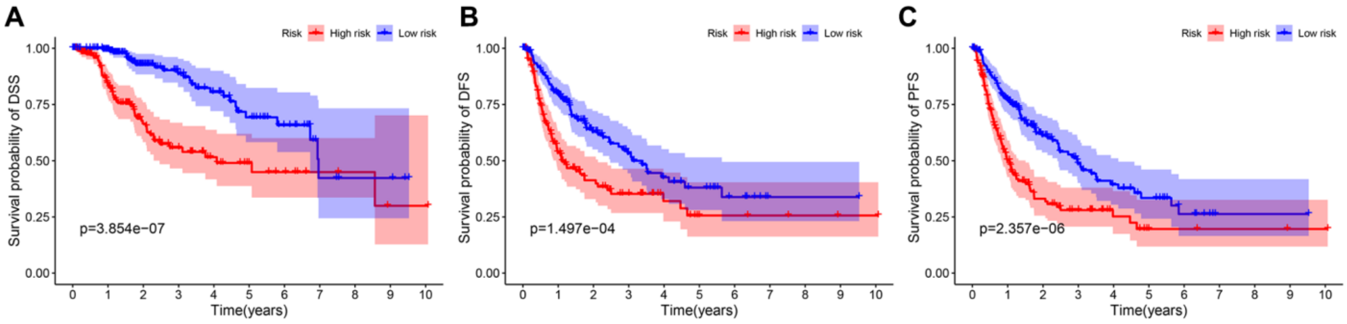

Supplementary Figure 2. Prognostic analysis of the 7-gene signature in the TCGA cohort. Kaplan-Meier curves for the difference in disease-specific survival (**A**), disease-free survival (**B**) and progression-free survival (**C**) of HCC patients between the high-risk group and low-risk group in the TCGA cohort.

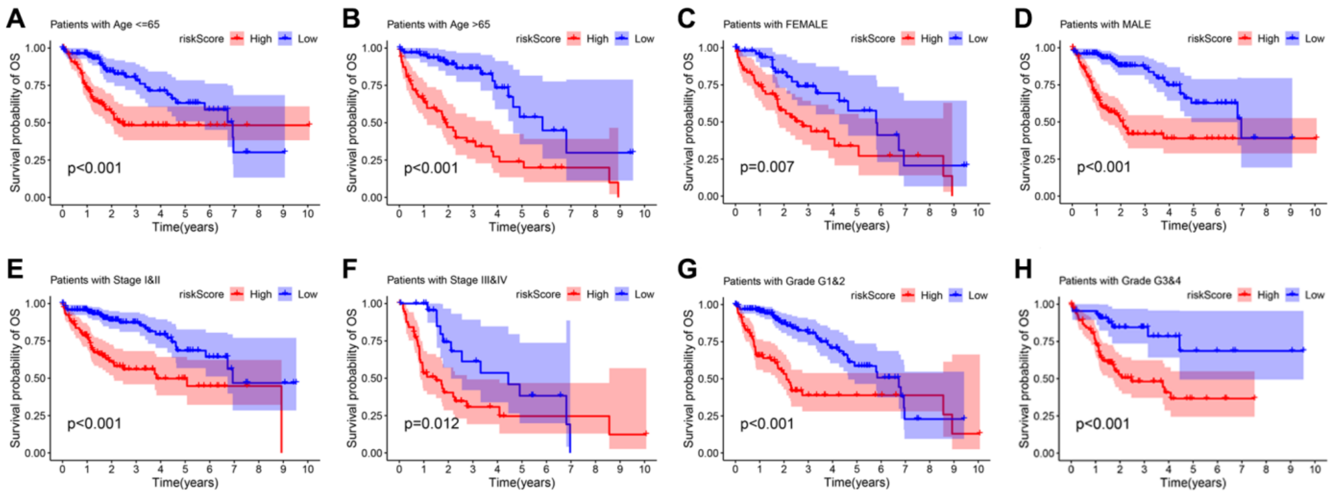

Supplementary Figure 3. Survival analysis of overall survival in each clinical subgroup in the TCGA cohort. The survival analysis of each subgroup has significant difference: age ≤65 (**A**) and age >65 (**B**), female (**C**) and male (**D**), stage I & II (**E**) and stage III & IV (**F**), grade G1 & 2 (**G**) and G3 & 4 (**H**).

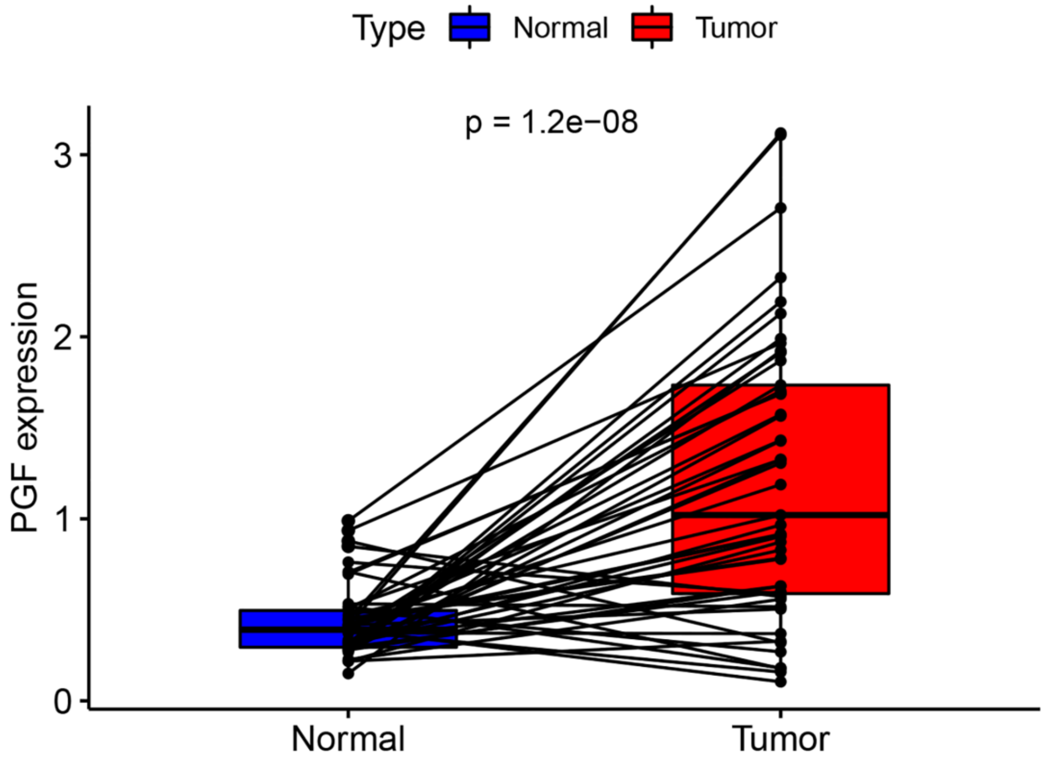

Supplementary Figure 4. The paired expression of PGF in the same individual normal tissue and tumor tissue.

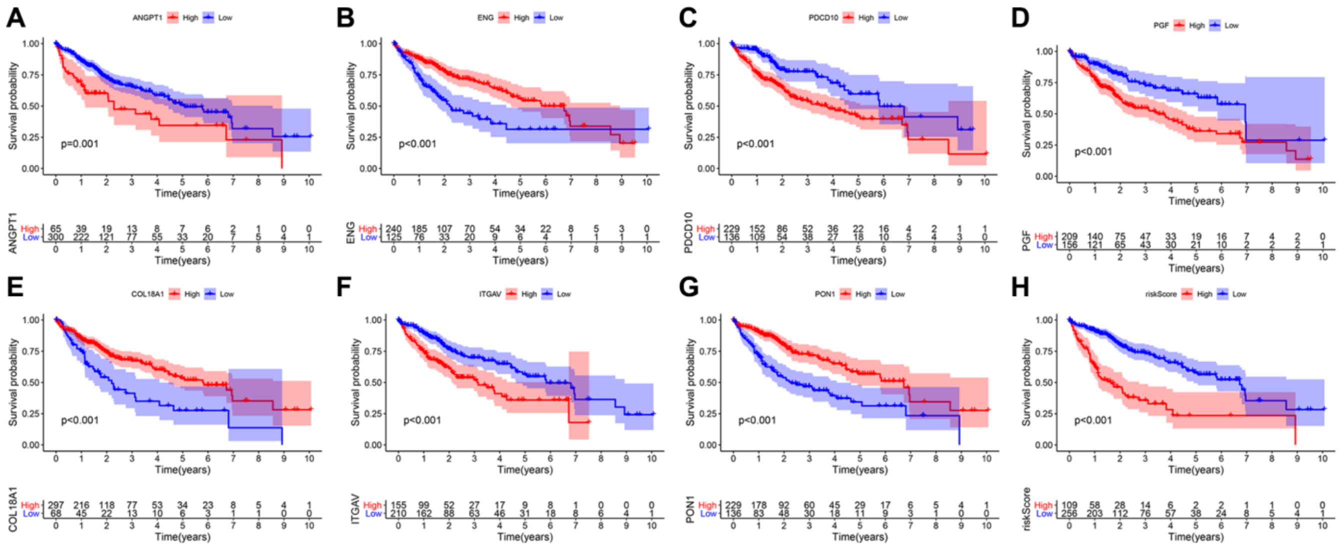

Supplementary Figure 5. Survival analyses according to the optimal cut-off expression value of each gene and risk score in the TCGA cohort. The expression difference of each gene (**A**–**G**) and risk score (**H**) are closely related to the prognosis of HCC patients in the TCGA cohort. All *P* < 0.05.

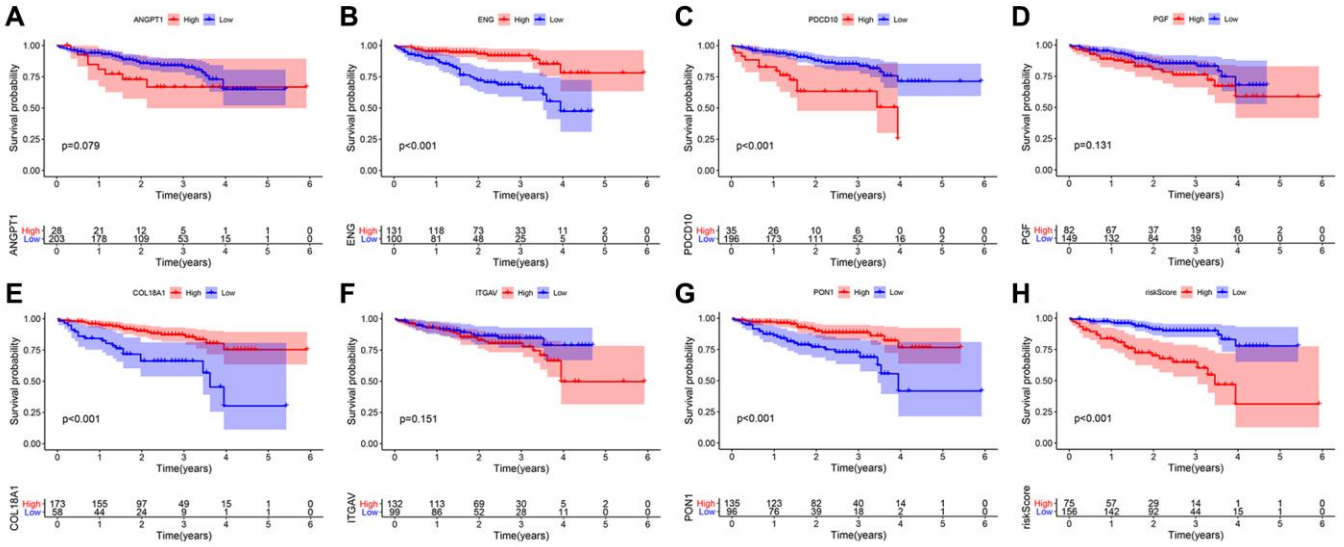

Supplementary Figure 6. Survival analyses grouped by the optimal cut-off expression value of each gene and risk score in the ICGC cohort. The expression difference of ENG (**B**), PDCD10 (**C**), COL18A1 (**E**), PON1 (**G**) and risk score (**H**) are closely related to the prognosis of HCC patients in the ICGC cohort. But ANGPT1 (**A**), PGF (**D**), and ITGAV (**F**) do not affect the prognosis.

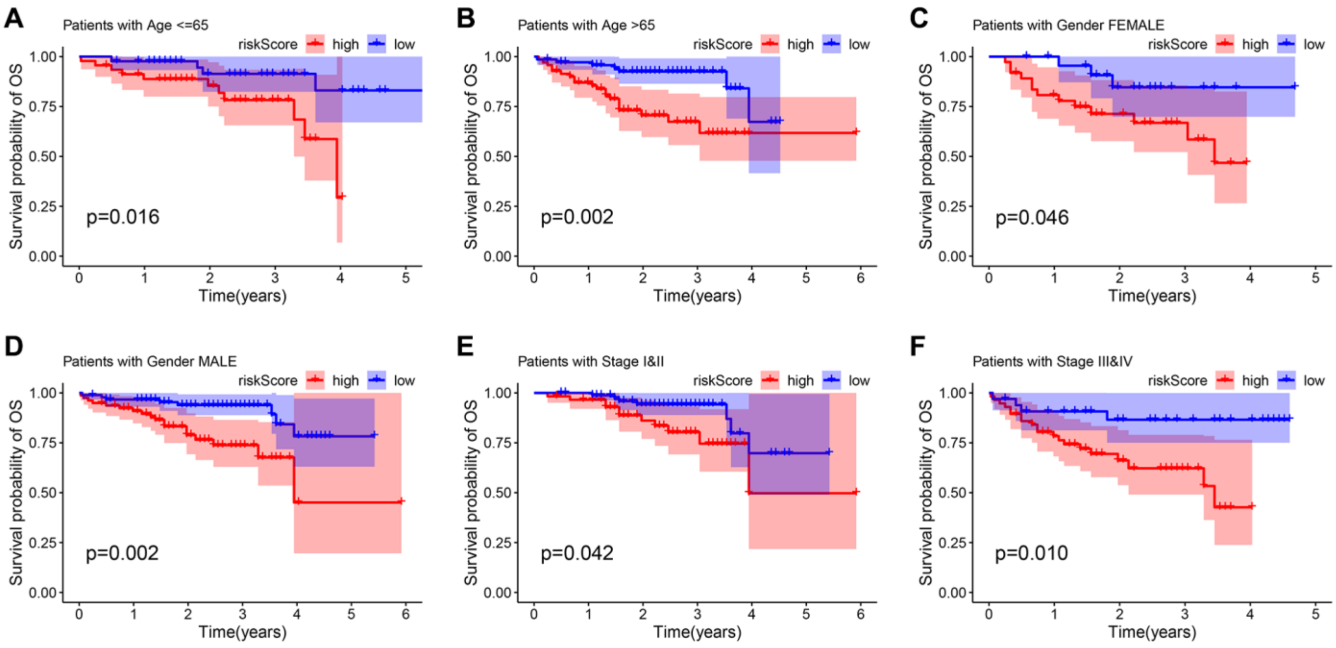

Supplementary Figure 7. Survival analysis of overall survival in each clinical subgroup in the ICGC cohort. The survival analysis of each subgroup has significant difference: age ≤65 (**A**) and age >65 (**B**), female (**C**) and male (**D**), stage I & II (**E**) and stage III & IV (**F**).
