# Supplementary material for: Screening and identification of angiogenesis-related genes as potential novel prognostic biomarkers of hepatocellular carcinoma through bioinformatics analysis
Source: Aging (Albany NY). 2021 Jul 12;13(13):17707–33. doi: 10.18632/aging.203260 (PMC8312452; doi:10.18632/aging.203260)
Supplement: Supplementary Tables 1 and 2 [file aging-13-203260-s002.pdf]

SUPPLEMENTARY TABLES

Supplementary Table 1. 52 differentially expressed angiogenesis-related genes.

Gene	log2FC	*P*-value	FDR	Gene	log2FC	*P*-value	FDR
**VEGFA**	0.838496	1.07E-07	2.18E-07	**TIE1**	0.672592	3.38E-05	5.75E-05
**TEK**	–0.76866	9.25E-11	2.91E-10	**VEGFD**	3.201947	1.79E-06	3.32E-06
**FLT1**	1.027421	2.78E-10	7.97E-10	**TGFB1**	1.303598	0.00252	0.003692
**ANGPT1**	1.547875	3.95E-08	8.29E-08	**CTNNB1**	0.742215	9.98E-12	3.70E-11
**ANGPT2**	1.734857	2.11E-14	9.50E-14	**VEGFB**	1.318205	5.30E-05	8.78E-05
**THBS1**	–1.01657	1.49E-09	3.77E-09	**CCM2**	0.260836	0.007736	0.01083
**VEGFC**	0.817592	1.08E-05	1.89E-05	**LRP5**	0.336904	0.01017	0.013632
**PLG**	–1.15095	1.74E-19	2.74E-18	**TYMP**	1.023739	1.52E-06	2.90E-06
**FLT4**	0.513711	0.007215	0.010331	**PTGS2**	–1.70841	5.31E-17	3.04E-16
**ENG**	–0.12314	0.018402	0.02366	**ITGAV**	1.428586	7.54E-09	1.76E-08
**MMP9**	2.720729	2.14E-08	4.65E-08	**IL1RN**	–1.51967	2.19E-11	7.27E-11
**KRIT1**	0.744827	1.25E-09	3.29E-09	**CCL2**	–0.70763	5.47E-07	1.08E-06
**PDCD10**	0.850463	4.09E-17	2.57E-16	**PDGFB**	1.568964	1.84E-18	2.32E-17
**NRP1**	0.790259	0.000261	0.000391	**EPHB4**	0.975849	9.66E-09	2.17E-08
**PGF**	1.843288	3.66E-13	1.44E-12	**PIK3CA**	0.305587	0.039747	0.048155
**AGGF1**	0.794556	1.14E-17	1.19E-16	**ITGB1**	0.770908	6.33E-05	0.000102
**HGF**	–1.38493	3.52E-20	7.40E-19	**KRAS**	0.405084	0.023271	0.028747
**PECAM1**	1.268413	1.39E-17	1.25E-16	**HFE**	0.799958	7.17E-09	1.74E-08
**DLL4**	1.756678	9.02E-22	2.84E-20	**TP53**	0.73094	5.80E-06	1.04E-05
**ADGRB2**	3.764124	2.96E-10	8.11E-10	**CXCL12**	–2.05117	1.08E-24	6.83E-23
**ADGRB3**	–1.12228	1.90E-17	1.50E-16	**AGTR1**	–0.82052	2.13E-13	8.96E-13
**FZD4**	0.436416	0.009991	0.013632	**PDGFRB**	1.823863	7.33E-17	3.85E-16
**ACE**	1.63276	1.28E-15	6.21E-15	**PON1**	–0.83373	1.72E-11	6.01E-11
**COL18A1**	–0.17402	0.010686	0.014026	**NOTCH1**	0.824596	0.000101	0.000156
**CDH5**	0.423801	0.018958	0.023887	**VWF**	2.050686	2.34E-17	1.64E-16
**TGFB2**	2.008395	9.02E-05	0.000142	**IL1B**	–1.06356	2.07E-10	6.22E-10

Supplementary Table 2. 79 angiogenesis-related genes retrieved from the GeneCards (Score >10).

Symbol	Description	Score
**VEGFA**	Vascular Endothelial Growth Factor A	62.61
**TEK**	TEK Receptor Tyrosine Kinase	37.41
**KDR**	Kinase Insert Domain Receptor	36.9
**FLT1**	Fms Related Receptor Tyrosine Kinase 1	32.35
**FGF2**	Fibroblast Growth Factor 2	31.09
**HIF1A**	Hypoxia Inducible Factor 1 Subunit Alpha	29.97
**ANGPT1**	Angiopoietin 1	27.43
**ANGPT2**	Angiopoietin 2	23.44
**ADGRB1**	Adhesion G Protein-Coupled Receptor B1	22.53
**THBS1**	Thrombospondin 1	21.51

**VEGFC**	Vascular Endothelial Growth Factor C	19.82
**AKT1**	AKT Serine/Threonine Kinase 1	19.41
**PLG**	Plasminogen	19.25
**FLT4**	Fms Related Receptor Tyrosine Kinase 4	19.19
**MMP2**	Matrix Metallopeptidase 2	19.16
**EPO**	Erythropoietin	19.04
**ENG**	Endoglin	18.94
**MMP9**	Matrix Metallopeptidase 9	18.79
**NOS3**	Nitric Oxide Synthase 3	18.74
**KRIT1**	KRIT1 Ankyrin Repeat Containing	18.52
**PDCD10**	Programmed Cell Death 10	18.38
**NRP1**	Neuropilin 1	18.03
**SERPINF1**	Serpin Family F Member 1	17.75
**PGF**	Placental Growth Factor	17.72
**AGGF1**	Angiogenic Factor With G-Patch And FHA Domains 1	17.44
**HGF**	Hepatocyte Growth Factor	17.07
**CXCL8**	C-X-C Motif Chemokine Ligand 8	16.89
**PECAM1**	Platelet And Endothelial Cell Adhesion Molecule 1	16.84
**DLL4**	Delta Like Canonical Notch Ligand 4	16.84
**NDP**	Norrin Cystine Knot Growth Factor NDP	16.61
**ADGRB2**	Adhesion G Protein-Coupled Receptor B2	16.61
**ADGRB3**	Adhesion G Protein-Coupled Receptor B3	16.16
**FZD4**	Frizzled Class Receptor 4	15.8
**ACE**	Angiotensin I Converting Enzyme	15.76
**COL18A1**	Collagen Type XVIII Alpha 1 Chain	15.48
**CDH5**	Cadherin 5	15.17
**TGFB2**	Transforming Growth Factor Beta 2	15.11
**SOD2**	Superoxide Dismutase 2	15.09
**MIR21**	MicroRNA 21	14.97
**TIE1**	Tyrosine Kinase With Immunoglobulin Like And EGF Like Domains 1	14.58
**VEGFD**	Vascular Endothelial Growth Factor D	14.32
**TGFB1**	Transforming Growth Factor Beta 1	14.23
**CTNNB1**	Catenin Beta 1	14.23
**VEGFB**	Vascular Endothelial Growth Factor B	13.66
**CCM2**	CCM2 Scaffold Protein	13.57
**LRP5**	LDL Receptor Related Protein 5	13.28
**TIMP2**	TIMP Metallopeptidase Inhibitor 2	13.25
**TYMP**	Thymidine Phosphorylase	13.13
**PTGS2**	Prostaglandin-Endoperoxide Synthase 2	13.02
**ITGAV**	Integrin Subunit Alpha V	12.73
**TIMP1**	TIMP Metallopeptidase Inhibitor 1	12.41
**VTN**	Vitronectin	12.12
**IL1RN**	Interleukin 1 Receptor Antagonist	11.82
**CCL2**	C-C Motif Chemokine Ligand 2	11.78
**PDGFB**	Platelet Derived Growth Factor Subunit B	11.71
**TIMP3**	TIMP Metallopeptidase Inhibitor 3	11.52
**FGF1**	Fibroblast Growth Factor 1	11.52
**HPSE**	Heparanase	11.44

**EPHB4**	EPH Receptor B4	11.27
**PIK3CA**	Phosphatidylinositol-4,5-Bisphosphate 3-Kinase Catalytic Subunit Alpha	11.12
**ITGB1**	Integrin Subunit Beta 1	11.03
**FGFR2**	Fibroblast Growth Factor Receptor 2	11
**CCN2**	Cellular Communication Network Factor 2	10.99
**KRAS**	KRAS Proto-Oncogene, GTPase	10.9
**BAIAP2**	BAR/IMD Domain Containing Adaptor Protein 2	10.84
**HFE**	Homeostatic Iron Regulator	10.81
**TP53**	Tumor Protein P53	10.73
**ETS1**	ETS Proto-Oncogene 1, Transcription Factor	10.71
**CXCL12**	C-X-C Motif Chemokine Ligand 12	10.69
**AGTR1**	Angiotensin II Receptor Type 1	10.63
**PDGFRB**	Platelet Derived Growth Factor Receptor Beta	10.61
**NRP2**	Neuropilin 2	10.55
**PON1**	Paraoxonase 1	10.52
**NOTCH1**	Notch Receptor 1	10.52
**ISM1**	Isthmin 1	10.39
**VWF**	Von Willebrand Factor	10.27
**BMP6**	Bone Morphogenetic Protein 6	10.27
**ITGB3**	Integrin Subunit Beta 3	10.22
**IL1B**	Interleukin 1 Beta	10.09
